# Supplemental Review of Practical Medicine; (for First Quarter of 1820) Selected and Arranged, with Commentaries

**Published:** 1820-04-01

**Authors:** 


### Supplemental Review

OF

### PRACTICAL MEDICINE;

( For First Quarter of 1820 )

SELECTED AND ARRANGED, WITH COMMENTARIES.

*Paucis libris immorari et innutriri oportet, si velis aliquid trahere, quod in animo fideliter hæreat.* SENECA.

Duo vitia vitanda sunt in cognitionis et scientiæ studio. ***** Alterum est vitium, quod quidam nimis magnam operam conferunt in res obscuras atque difficiles, easdemque non necessarias. CICERO.

THE leading policy of the Medico-Chirurgical Journal is the segregation of what is really useful and practical in medical literature and science, from that which is hypothetical, erroneous, or merely curious. And it is upon the judgment with which this appreciation is made, and the soundness of the principles conveyed in the running commentaries, that we ground our claims to public patronage. Having ourselves passed through most of the stations in which are placed the great majority of our readers, we know their wants, and their wishes. We therefore confidently appeal to them, whether or not a Journal was wanting, which, by confining itself to those objects that were more immediately of a *practical* nature, might thereby render itself more extensively useful to the great body of the profession, than if it indulged in diffuse criticisms, elaborate researches, and erudite disquisitions. The palm of superiority, in these last respects, we freely and unaffectedly resign to those of our cotemporaries who covet and merit it, leaving it to the public to approve or condemn the line of policy and conduct which we have here embraced.

The present article is designed as an improvement on what are termed "Retrospects" of medical science; at least it is a modification of such, in accordance with the principles and spirit of a Journal which rejects all the incomprehensible, and almost unutterable mysticism of German incubation, together with the endless record of nameless nothings, as tending only to render the human mind and memory a chaos of cabinet speculations and discordant elements, instead of a granary of clinical facts and practical deductions. The time of our readers and the pages of this Journal are too limited, and too valuable, to be squandered away in the pursuit of chaff, which flits about with every breeze of hypothesis, while the solid and useful grain is under our feet.

In this Supplemental Review then, we shall be exceedingly fastidious in the choice of our selected materials, choosing rather to leave some good behind, than bring any thing useless away. To avoid all tautology too, we shall rarely touch upon those works which are designed for regular analysis in separate articles; and, as a standing principle of our policy, we shall seldom occupy space with mere references and title pages, unaccompanied by substantial and useful matter. We purposely adopt this plan, because it is one which has not been adopted elsewhere; and we do not choose to follow (even had we leisure for that purpose) the steps of others, when we can pursue an original path of our own. We may take this opportunity however, to acknowledge that the literary labours of our cotemporary Journalists are highly creditable to their talents and acquirements; nor do we deviate from the laudable and erudite course which they pursue, through vanity or disrespect, but from a thorough conviction that, by limiting the circle of our research, and carefully exercising the prerogative of our judgement, we shall fill up a hiatus in the periodical literature of the day, and furnish a desideratum of no mean value to the general mass of medical practitioners.*

It only remains to say a few words in respect to the arrangement of this article. We shall follow, in some mea-

---

* To the list of our respected cotemporaries in this metropolis, The London Medical and Physical Journal; The London Medical Repository; and the Quarterly Journal of Foreign Medicine and Surgery, another interesting Monthly Publication has recently been added, which has our entire approbation, and merits the notice of the profession at large. The Medical Intelligencer, giving a short abstract or analysis of all the medical articles published throughout the whole circle of the periodical press, condenses into a single monthly sheet, price only 8d, a most astonishing mass of select and valuable matter. Such is now the extent of periodical publications, and the quantum of medical articles scattered through them, that an Indicator or Collector of this kind was greatly wanted, and will prove a most useful work. The Editor appears to possess a philosophic mind, ingenuous candour, and an inflexible integrity. With these qualifications, and a proper share of industry, his Intelligencer must succeed.

sure, the *order* which Morgagni and Bonetus observed in their pathological writings; an order, which, at once, disembarrasses us of all nosological distinctions and hypothetical subtleties. We shall class our subjects as they appertain to, 1st, the head; 2d, the neck; 3d, the thorax; 4th, the abdomen, including the pelvis and its outlets; 5th, the extremities; 6th, the skin; 7th, the nervous system; 8th, the vascular system; 9th, the absorbent system; and, 10th, the general system, or the greater part of it. In this arrangement, Medicine and Surgery must be blended, as they often are; but chemical and pharmaceutical subjects will come in at the end, if they cannot be elsewhere introduced with propriety, under the *title* of "medicinal agents."

——— Si quid novisti rectius istis,
Candidus imperti; — si non, his utere mecum.

---

### § I. HEAD.

I. *Cephalea.** A young woman, ætat. 24, had been afflicted with constant beating pain in the left cheek and upper jaw, along the alveolar process, stretching to the throat, "and, indeed, involving the whole head." The pain was without respite, and so violent, that she constantly supported her head, unable to use the slightest exertion, and sleeping only when completely exhausted. "Of course, all kinds of remedies had been tried, external and internal." This is a very vague expression, especially when Mr. Liston solicits suggestions respecting the treatment which has hitherto been unsuccessful. Who can prescribe, after "all kinds of remedies" have been used in vain. But the fact is, that all kinds of remedies had *not* been tried, for Mr. Liston himself put in practice a new one; namely, the ligature of the carotid artery of that side. He was naturally enough led to this experiment, by finding that pressure with the thumb upon that vessel quickly mitigated all her sufferings. For some time after the operation, the patient was much relieved; but since then, the pains have returned with violence.

We have often had occasion to observe the erroneous importance which is attached to the *vis a tergo*, or, as Dr. Parry expresses it, the momentum of the circulation. When a tissue, or structure of any kind, is in a state of irritation, inflammation, distention, or organic lesion, every impulse of blood from the heart, in general, augments the pain. But where numerous channels lead to the seat of lesion, it is in vain to look for permanent relief from the obliteration of any one. The vascular distention is again reproduced through

---

* By Robert Liston, Esq. &c. &c. Ed. Journal, 62.

other circuits. The object is clearly, according to our apprehension, to reduce the whole circulating mass ; to keep it in a state of reduction proportioned to the local lesion ; and, during that time, to endeavour to restore the healthy state of the part itself. This must be done by local and general blood-letting, rigid abstinence, constant action of the bowels, and quietude. In short, not only should the balance of the circulation, but that of the nervous excitement, be diverted, if possible, to another quarter than the head; especially to the intestinal canal — *qua data porta.* We know very well that it requires great confidence in the physician to have these measures rigidly, and for a sufficient time, put in execution ; but this much we can assure Mr. Liston, that by an inflexible and persevering course of the foregoing measures, we have overcome diseases, after " all kinds of remedies had been tried," with no kind of energy or effect.

II. *Ophthalmia.** The continental physicians have been much more attentive to those modifications of inflammation resulting from the nature of the texture invaded, or the constitutional disease of which the inflammation is the local demonstration, than we have been. The attention of British pathologists, however, is now directed to this investigation ; and we have no doubt of a beneficial result.

1. *Description.* Mr. Wardrop observes, that in the *Rheumatic* Ophthalmia, the red colour of the *albuginea* is not the bright crimson accompanying inflamed *cornea;* nor is the redness confined to one spot, as in pustular ophthalmia. Neither is there the puriform discharge attending inflamed conjunctiva. The albuginea acquires a brick-red tinge, or an admixture of yellow with crimson red. The blood-vessels are generally equally numerous over the whole white of the eye, passing forward in nearly straight lines from the posterior part of the eye-ball, and advancing close to the cornea; but neither passing over it, nor leaving the pale circle around it, which is so striking when either the choroid coat or iris is inflamed. As the disease advances, the cornea becomes dull and turbid, sometimes with a general cloudiness, more opake in the centre than circumference. If minutely examined, some abrasions of the cornea and conjunctiva will be detected. In the beginning there is a dryness of the eye, succeeded, sooner or later, by a very copious lachrymal secretion. The eyelids in this disease are but slightly swelled, and their vascularity little increassd. The chief seat of pain, at the commencement, is in the head, sometimes affecting the eye-ball itself, or extending to the temple, cheek-bone, teeth, or lower jaw. Occasionally there is precise hemicrania, of a dull agonizing, rather than an acute kind, and coming on at times in severe paroxysms. Sometimes the pain is excited by merely touching the scalp ; it is

* An Account of the Rheumatic Inflammation of the Eye, with Observations on the Treatment of the Diseases. By JAMES WARDROP, Esq. F. R. S. E.—Medico-Chirurgical Transactions, vol. x.

usually remittent, coming on at four, six, or eight o'clock in the evening, continuing all night, being most severe at midnight, and becoming abated towards morning. The eye is not very morbidly sensible to light in rheumatic ophthalmia.

Our author considers the seat of this affection to be the sclerotic coat chiefly, which belongs to the fibrous tissues, the favourite domiciles of rheumatism.

To the local symptoms there is generally superadded pyrexia, with much functional disorder of the primæ viæ, and vitiation of the secretions and excretions. The progress and severity of the disease are very various; the attack being sometimes slight and transitory, leaving no permanent injury of the eye. At others, long and severe, ultimately destroying the visual organ, by ulceration of the cornea bursting forth of the aqueous humour, or puriform secretion in the posterior chamber of the eye.

In respect to etiology, rheumatic ophthalmia may, in many instances, be traced to sudden atmospherical transitions; is most frequent in the spring months, and often follows the operations for cataract, especially in patients of rheumatic tendency. It usually affects but one eye, sometimes both, the inflammation being seldom so severe in the second eye as in the one first affected.

The rheumatic resembles the syphilitic more than any other ophthalmia, and Mr. Wardrop has frequently seen them confounded; yet he thinks they may be distinguished from one another by the appearances of the inflammations, the progress of the diseases; but above all, by the constitutional symptoms which accompany them.

"In the rheumatic inflammation, says Mr. W. it has already been noticed that the proper vessels of the sclerotic coat are enlarged, which is the cause of the redness being generally diffused over the whole albuginea; whereas, in the syphilitic inflammation it is the anterior ciliary arteries passing along the sclerotica on their way to the iris, which are chiefly affected; and thus [hence] the pale ring which is always observed between the cornea and enlarged vessels in that disease." 10.

Those too who have the syphilitic inflammation in the eye have always, our author avers, the constitutional symptoms of syphilis, the history of which can be distinctly traced.

2. *Treatment.* In this species of ophthalmia Mr. W. has found evacuation of the aqueous humour advantageous; especially where proper means had been neglected in the beginning; where there was attendant pain; where the cornea had become dim and clouded; and where the vision was impaired. In all such cases, the good effects of the operation were instantaneous; after which, he found no other applications necessary, than fomentations to the parts around the eye, and, where the eye itself remained irritable, some vinous tincture of opium.

The digestive organs, in this complaint, as indeed in most others, require great attention. Mr. W. has seen much relief obtained from the exhibition of an emetic, composed of vinum ipecacuanhæ one ounce, and a drachm of the liq. antimon. tart. After the emetic the bowels should be completely evacuated; "and a couple of grains of calomel combined with a few of rhubarb may be two or three times given; also saline, or other purgatives, if necessary, with a view to act on the biliary organs, as well as empty the alimentary canal." 12.

When this inflammation has succeeded a sudden chill, the functions of the skin should be restored by the judicious employment of sudorific medicines. "A couple of grains of antimonial powder given singly, or combined with opium, every four or six hours, is an excellent remedy," often allaying the evening paroxysm, when exhibited a little before the accession. Our author has seen little advantage derived from local or general bleeding, unless there was tendency to plethora, with a very full and hard pulse, when one or other of the evacuations would be proper. But, in general, "the little relief afforded by bleeding in this disease, may be regarded as one of its diagnostic characters." Fomentations and blisters, repeatedly applied behind the ears, or to the nape of the neck, are useful auxiliaries. "The vinous tincture of opium is the only local application which he has ever observed to be decidedly beneficial; but its use should always be deferred till the latter period of the inflammation, when all febrile symptoms are subdued." A small quantity of this fluid may be put within the eye-lids once or twice a day, with a common camel's hair pencil, and its use persisted in while it affords relief.

If, after the *primæ viæ* have been well evacuated, the tongue still remain very white, with quickened pulse, Mr. W. has seen decided benefit from *cinchona*, singly, or combined with the mineral acids. It should only be given in small doses, generally from five to eight grains in a little warm water every two hours.

Mr. Wardrop was led to this treatment from the remarkable effects which he had observed, in the cure of rheumatism of the joints, from cinchona. Mr. W. appears to think bark as complete a specific in this disease, as in ague; but we fear that the generality of the profession have not been so fortunate in finding a specific for rheumatism. The paper before us is very creditable to Mr. Wardrop's well-known talent for acute observation, and felicity as well as facility of description.

While on the subject of ocular inflammation, we may here notice an observation of Mr. Guthrie, in his recent publication on artificial pupil.* It is well known that in idiopathic iritis, blood-letting and mercury have been found the paramount measures of cure. Mr. Guthrie, in the work above mentioned, informs the profession, that, in the iritis which occasionally supervenes on the operation for artificial pupil, mercury is equally efficacious as in the idiopathic, or, as

---

* A Treatise on the Operations for the Formation of Artificial Pupil, in which the morbid States of the Eye requiring them are considered, &c. &c. By G. J. Guthrie, &c. &c. &c. with Plates. Octavo, pp. 210, London, 1820.

some have supposed, the syphilitic forms of the disease. "The great utility of mercury in inflammation of the iris resulting from wounds, has not, I believe, been either noticed by authors, or generally understood. It is not, however, more valuable in the one case (idiopathic) than in the other, (the traumatic), and the use of it should never be neglected, when bleeding is insufficient for the suppression of the inflammation, which will, in all probability, prove destructive to vision." *Treatise*, p. 157.

III. *Ophthalmia Periodica.** We should conceive that the *patient* in this case was also the *physician.*—J. B. ætat. 46, spare, and delicate habit, yet active, and without any hereditary disposition to disease, but subject to stomach complaints, becomes affected, in the month of June, every year, with a greater or less degree of the following symptoms: A sensation of heat and fulness in the eyes, with, at first, a slight degree of redness, and discharge of tears, gradually increasing until the sensation becomes converted into a combination of the most acute itching and smarting, with a feeling as though a number of small points were darting into the eye-ball; the eye at this time becoming inflamed, and discharging copiously a thick mucous fluid. This state of the eyes comes on in paroxysms, with uncertain intervals, from about the middle of June till the middle of July; the violent paroxyms never occurring more than two or three times daily, lasting an hour or two each time. The paroxysms may generally be traced to certain exciting causes, as a close moist heat, a bright glare of light, dust, and other substances stimulating the eyes. After these ocular phenomena have continued for a week or ten days, a general fulness is experienced in the head, to which succeeds irritation in the nose, with strong paroxysms of sneezing. To these are soon added a sensation of tightness of the chest, and difficulty of breathing, with a general irritation in the fauces and trachea, but without any absolute pain in any part of the chest. To these local symptoms are added, a degree of general indisposition; languor; incapacity for muscular exertion; inappetency; emaciation; restless nights; often attended with profuse perspirations; the extremities, however, being generally cold; the pulse permanently quickened, from 80 to 100 or 120.

The above may be considered a severe attack; and after such, the patient has generally better health than usual in the autumn. Almost every kind of medicine has been tried, with little or no benefit. "By using every means of obtaining fresh air, without much exertion, and by carefully avoiding a moist and close atmosphere, the symptoms may, in some measure, be kept off; but they have frequently appeared under circumstances that seemed the least likely to have produced them."

---

* Case of a Periodical Affection of the Eyes and Chest. By John Bostock, M. D.—Med. Chir. Transactions, vol. x.

We have not the smallest doubt that the above complaint is one of those anomalous forms which irregular gout masks itself under, in particular constitutions. We have seen a case very similar to the above, only much more frequent in repetition, and severe in degree, in a female. We tried a great many remedies, and at length some rough ones. The disease disappeared for a time, but alarming symptoms of organic affection of the womb succeeded. The old complaint once more became reinstated, and the uterine symptoms instantly gave way. Barthez and Guilbert relate instances somewhat analogous to the above, where concealed gout played upon various tissues and organs in the most unaccountable shapes. We would therefore advise the narrator (if he be the patient) to *respect* the present manifestation of the complaint, and to rest contented with a mitigation of the malady by the prophylactic measures which he has found useful, rather than aim at the entire removal by *medicine*, of so long established a train of phenomena in the system, at the risk of incurring other and more dangerous affections.

IV. *Hemerailopia.** Mr. Simpson distributes his work into eight sections; the outlines of which are, preliminary observations on the structure and functions of the eye; history and definition of the disease, with critical remarks on its classification; theory of its causes and nature; its diagnosis; its prognosis; its treatment; miscellaneous animadversions upon the peculiarity of the antecedents commonly necessary for its production, and on its combination with other complaints; practical illustrations, with commentaries on the cases.

Hemerailopia is preferred by Mr. S. to all other terms, as a descriptive appellation for nocturnal blindness. How a term implying only *vision by day* can come to be the one most expressive of *blindness by night*, is a problem whose solution, in our opinion, is not so very obvious. Give us, however, a good definition of *things*, and we shall not be disposed to cavil about *names*. Our author allows this disease to remain, for the present, in Dr. Cullen's genus *Dysopia*, although there seems to be a "relationship between it and Amaurosis, which, in the existing condition of medical science, is not conveniently determinable." His views of its nature and treatment are founded on "autoptical" observations made in tropical regions and at sea.

Mr. S. is inclined to regard Hemerailopia as a symptomatic disease nearly peculiar to intertropical climates, and as originating, in most instances, from a scorbutic predisposition connected with a state of vascular congestion in the eyes themselves, and in the anterior lobes of the brain. He considers its diagnosis as being sufficiently perspicuous; from amaurosis, "it is conveniently distin-

---

* Observations on Hemerailopia, or Nocturnal Blindness; with Cases and Practical Illustrations. By ANDREW SIMPSON, Surgeon. Octavo, Glasgow, 1819, pp. 132.

guishable by the patient's capability of seeing in the day-time, and his dimness or imperfection of vision returning (p. 47) as the vespertine obscurence approaches." The prognosis, he says, should in general be favourable. By his own "Illustrations," however, Mr. S. exhibits evidence in favour of dissolution or recovery being the consequence, on an equal scale.

Mr. S. very properly recommends particular attention to the state of the gastric, hepatic, and encephalic functions, as the basis of a general treatment. He advises cautious and moderate purgation, with occasional vomiting; and, for these purposes, he employs arbitrary modifications of the common means, assisted by dietetical regimen, vegetable acids, *astringents*, *tonics*, and ablutions with warm salt-water. His topical remedies consist of errhines, alkaline vapours, and blisters. He denounces general blood-letting as inadmissable, and is doubtful how far the use of leeches may be serviceable. Leeches and the lancet, he acknowledges, have never been employed by him in treating the disease. According to his own theory of its nature, topical bleeding, especially by arteriotomy, is in our opinion clearly indicated; and the surgeon who allows himself, at the out-set, to be deterred by *scorbutic debility* from a prompt and free employment of sanguineous depletion, together with emetics and active purging, will certainly lose an opportunity of benefiting his patient, which he may never afterwards regain. If vascular congestion in an organ do not require detraction of blood from the vessels which feed that organ, we confess our incapacity to define a case wherein the practice can ever be indicated. Instead of five grains of calomel, we apprehend, moreover, that a few doses, each consisting of four or five times that quantity, will be found to be productive of more advantage in *symptomatic* hemerailopia than all the complicated prescriptions wherewith the pages of this volume are so liberally adorned.

Four elaborate cases, and one, " given (p. 125) without pathological considerations,' are detailed in illustration of the author's doctrines. In two, the patients recovered; in two, they died; in the *odd* one, " the ocular ailment (p. 132) was seemingly removed." There was no dissection.

We have not been able, on a close examination of this volume, to discover any thing in it which had not already obtained the notice of previous observers. It may not perhaps be unprofitable in conclusion, to present Mr. S. and our readers with a summary view of the opinions which regulate our practice in this unfrequent disease. —Hemerailopia, then, is a morbid state of the eye, wherein vision is obscure in the morning, perfect at mid day, defective towards evening, and suppressed during the night. It is as much an idiopathic affection as any other, and depends more immediately on imperfect action of the retina. Any cause capable of embarrassing the functions of the optic nerve, by inducing congestion in the ophthalmic vessels, or by intercepting the diffusion of sentient energy in that nerve itself, possesses the power of establishing that imperfect action of the retina by which nocturnal blindness is determined.

Such a cause may occasionally have place in any region of the habitable earth. The periodical character of this malady may be referred to that peculiarity of circumstances from which the intermissions of disease is at all times to be retraced. We have never failed to remove congestive hemerailopia by practising arteriotomy freely in the temples, and assisting its operations with a large proportion of the submuriate of quicksilver. Nervous hemerailopia will, in nine cases out of ten, yield to the use of topical blisters or setons, emetics, and nauseating cathartics, administered with firmness and circumspection.

At parting, Mr. S. in whom we would recognize the traits of a generous mind, will permit our objecting to his "Tractate," as being, in many places, loaded with "complicated and cacophonous appellations," whereby it is made as defective in perspicuity as it is redundant in style.

V. *Teeth.** The importance which is now attached by the public to DENTAL pathology, is sufficiently evinced by the great segregation from the body of the profession, and the concentration of talent and respectability in this exclusive line of practice.

The general expression of public feeling would appear to indicate that division of labour in the medical, as well as in every other art, tends to accelerate its progress towards perfection. The senses of sight and hearing are now separately studied, and the diseases of them claimed by their respective cultivators; and it is highly probable that, ere a century elapses, we shall have the stomach, the lungs, the liver, the kidnies, and the brain, each the foundation of a distinct professorship, at least in the metropolis and the larger cities of the empire. Yet, when we consider how intimately the various internal organs are associated in function, and how necessary it is, that the whole body should be carefully studied, in order to comprehend the structure and office of a part, we are induced to discountenance this system of exclusive organology, since we are confident that it opens a wide field for the exercise of that wretched practice of *demi-charlatanism* which is the disgrace, the bane, and almost the ruin of our science! We have seen so much of this in our professional pilgrimage, especially in private practice, that we shall take an early opportunity of introducing some severe, but we hope, just strictures on certain disingenuous manœuvres, too often employed by men who ought to remember the dignity of the science they profess. These remarks are not levelled against any particular class of practitioners, but against the *trick of trade*, wherever it exists, and by whomsoever practised. In short, we consider that a neglect of the almost divine precept conveyed in the motto of our Journal, is the worst evil existing in the medical

* Observations on Diseases of the Teeth. By THOMAS BELL, Esq. F.L.S. Medico-Chirurgical Transactions, vol. x.

profession, and that which stands in the greatest need of reform. But more of this hereafter.

We believe with Mr. Bell, that the vital organization of the teeth is a generally received doctrine among those physiologists who have paid most attention to the subject; and the first fact related by our author is sufficiently demonstrative of the solid foundation of the said theory. A medical gentleman had suffered, for some time, the most excruciating pain, though not of the nature of common tooth-ache arising from caries. The alveolar process and gum had undergone absorption, so that the fangs were partly exposed; and although there was no external appearance of disease in the tooth, yet, as it was productive of so much pain, and had become loose and useless, Mr. Bell extracted it. He then sawed transversely the crown of the tooth, and found a cavity formed in the bony substance of it, communicating with the natural cavity, and filled with pus. "There was not the slightest appearance of that softness and discoloration which essentially characterize caries; on the contrary, the texture of the surrounding bone remained unchanged, and possessed a remarkably firm and healthy appearance." Mr. Bell conceives that there is but one way of accounting for this —namely, that it was a distinct abscess formed in the bone of the tooth. "The membrane had been inflamed, suppuration followed, and the pressure of the pus occasioned absorption. How could this take place in an unorganized structure?"

Our author believes, then, that inflammation and mechanical injury are the *only* causes of caries, and he thus differs from those who assert, that mere contact of a carious tooth occasions disease in one which was previously healthy. "The whole crown of the tooth, (says he) that part in which caries almost always commences, is completely enveloped in a covering of enamel, which is composed of phosphate of lime. Now as caries originates in the bony substance, it is clear, that for disease to be produced from any merely external cause, either chemical or mechanical, destruction of the enamel must first take place. But there is no agent evolved during the decomposition of a carious tooth, which can in any possible manner act upon the enamel; therefore, the mere contact of a decayed tooth cannot affect a healthy one." Yet, as we very frequently see caries in two contiguous teeth, it is desirable that the circumstance should be accounted for. He attributes it to one of two causes:—"In the first place, (says he) it is reasonable to conclude, that any cause producing inflammation and consequent caries in any one tooth, would, with equal probability, have the same effect on the one in contact with it; and as one may be expected to have suffered more severely than the other, it would the more speedily show symptoms of decay, and thus be supposed to *produce* the caries shortly after developed in its immediate neighbour." The other cause he attributes to the pressure of the two teeth against each other, by which the enamel of both becomes more or less mechanically broken down at the point of contact, and the bony substance more exposed to the action of the common causes of caries.

Mr. Bell relates a case illustrative of the effusion of coagulable lymph around the fang of a tooth, the consequence of severe inflammation, and the cause of very distressing symptoms. The following is a condensed analysis of the case.

A gentleman, after exposure to cold, became affected with intolerable pain, darting in paroxysms, from one of the molar teeth of the lower jaw, towards the ear. The tooth was loosened, and somewhat raised in the socket, and pressure so much augmented the pain, that mastication was impracticable. No appearance of disease in the substance of the tooth itself. On extraction, the lower part of the fang was found completely enveloped in a thick coating of adhesive matter, the result of intense inflammation. "In a few days, however, all the molares *of that side*, both in the upper and lower jaw, were affected in a similar manner, and to as great a degree as the one I had extracted; they were in the same way raised somewhat above their natural level in the jaw, and produced equally severe pain on pressure. The face was much swelled, the submaxillary glands enlarged, the gums highly inflamed, and the most violent pain extended along the jaws, behind the ear, and over the forehead." Mr. Bell, profiting by the appearances in the extracted tooth, scarified the gums freely, purged the patient briskly, and put him on abstemious diet. As there was not much relief from this plan, leeches were next day applied to the gums, external part of the cheek, and base of the lower jaw, with a blister behind the ear, the bowels being still freely acted on. Perseverance in this plan for a few days, totally removed the inflammation and its consequences. The teeth became firm, and the power of mastication was restored.

The above, Mr. B. thinks, may fairly be considered as constituting the first stage of those abscesses which occasionally form at the extremity of the fang of a tooth, and produce sometimes extensive disease of the alveolar processes and gums. If this be correct, the opinion that these abscesses arise *exclusively* from the irritation of a caries, or of a fang remaining as an extraneous body in the socket, must be given up; for in the case alluded to, not one of the teeth was, in the least degree, affected with caries.

Finally, it appears to Mr. Bell, that the diseases above mentioned, however they may differ in their *nature*, (we had rather say in their *results*) have but one cause—namely, INFLAMMATION; and that, were early attention paid to this circumstance, and decisive means had recourse to for its removal, much pain and subsequent disease might be prevented. The dense structure of the teeth prevents their yielding to the increased thickness of the lining membrane, when inflamed; and from their low degree of organization, the process of absorption is necessarily slow. Hence the great importance of preventing inflammation, or of quickly arresting its progress, when it has once commenced. Whatever produces pain in a tooth, generally speaking, is injurious; and the application of food or drink, of very high or very low temperature, is the most common cause of disease. We think, however, that Mr. Bell overlooks too much the action of the remains of food, in a decomposing state, on the enamel of the teeth, as a cause of caries, and the consequent necessity of removing all such extraneous matters from between them after meals, by means of ablution and the brush.*

We recommend Mr. Bell to pursue his investigations, as he appears to possess the requisite talents for conducting them with success.

---

### § II. NECK.

VI. *Laryngitis.*† There are few diseases more distressing in their appearance, or fatal in their consequences, than inflammation of the larynx. The delicate membrane lining the complicated structure of this part is exposed so constantly to atmospherical transitions, that it is wonderful the complaint is not more frequent, in this climate, than we actually find it. It has, of late years, however, pressed more on the attention of the faculty than formerly, and it is one where medical and surgical skill is eminently beneficial, when promptly applied.

Laryngitis acuta makes quick work with its victim; and unless the young practitioner's mind be already stored with the necessary resources upon such an occurrence, he stands a chance of losing his patient, and probably a portion of his reputation, which he may be ill able to spare during the first years of his professional progress. On this account, we shall take the present opportunity of introducing some practical observations on the disease in question, before we analyse the case at the head of this article.

Laryngitis is an inflammation of the mucous tissue lining the various parts which compose the interior of the larynx, the glottis, and epiglottis. The patient feels an acute pain in that part of the throat, on attempting to swallow or speak, with a stridulous voice, short dry cough, great difficulty of breathing, redness of the face, prominency of the eyes, and, of course, symptomatic fever. It may terminate in three or four different ways—in resolution, by prompt and decisive measures; in sudden death, by suffocation, from the tumefaction of the parts; in the complete disorganization of the mucous membrane of the larynx, by mortification; in an ulceration of some portion of the larynx, ending in laryngeal phthisis; or, finally, in a partial obliteration of the rima glottidis, of whieh we have stated a memorable instance in a former number of this Journal.‡

---

* Mr. Parmly, an ingenious and expert dentist of this metropolis, maintains, in his work and lectures on the Teeth, that external agents are the principal causes of decay, and his apparatus for removing all impurities from their surface, we have found extremely useful, and can therefore recommend it. Rev.

† A Case of Chronic Inflammation of the Larynx, in which Laryngotomy and Mercury were successfully employed. By Marshall Hall, M. D. of Nottingham.

‡ Vide, Page 119 of our first Number.

The exciting causes of this dangerous disease are almost invariably atmospherical transitions, or the application of cold or wet to some portion of the surface of the body, when hot or perspiring. We believe that a syphilitic taint in the constitution is rarely more than a predisposing cause of this affection.

If we see the patient before the inflammatory stage is past, which will generally be the case, (for people are not very slow in summoning the Doctor, when they find themselves threatened with suffocation) we must put in force, not in lingering succession, but in simultaneous co-operation, if possible, every means which medicine and surgery can suggest for the speedy reduction of this destructive inflammation. Every medical man, whether physician or surgeon, should carry a lancet in his pocket, and be ready to open a vein, on an emergency; for the College of Physicians have wisely imposed no oath against phlebotomy, as Hippocrates did against lythotomy. Whoever sees the patient first, then, in laryngitis, should instantly open the largest vein he can find in his arm, and abstract blood till *deliquium animi* suspends the flow, while leeches and blisters should be procured with all possible speed. Mean time, a large dose of calomel should be given, and followed by such other purgatives as are most ready at hand, or easily swallowed. As soon as the leeches arrive they are to be applied in clusters all over the throat, while a large blister is laid on, from the leeches, over half the sternum. During these transactions, the warm bath is to be preparing; and, as soon as ready, the patient is to be immersed up to the scrobiculus cordis in it, and bled again, if no alleviation followed the first venesection. The patient should be directed to breath, as much as possible, through the spout of a tea pot, containing warm water. When the purgatives have acted briskly on the whole line of the intestinal canal, the tartrite of antimony and digitalis should be exhibited so as to reduce the action of the heart, and produce nausea at the stomach. If these measures, reiterated as symptoms may require, do not reduce the inflammation, and relieve the breathing, we may prepare for bronchotomy, for no other means will save the life of our patient. By these means we believe that we have saved some lives; and in one case which, in a subsequent attack, required bronchotomy, we completely dissipated the most violent and acute laryngitis that had ever come in our way.*

But to the case before us. Mrs. Hatton, ætat. 53, became affected in September 1817, with hoarseness and hard dry cough, which continued to augment for two months. About the middle of November, a degree of difficulty of breathing, referred by the patient to a "tightness in the throat," was superadded. She now discovered that she was unable to "snuff up" through the nose in inspiration, in the ordinary way. All these symptoms went on in aggravation till the beginning of February, 1818, when a degree

* See Medico-Chirurgical Journal, (Monthly Series) Vol. V. page 1.

of dysphagia was experienced. In March, she observed a rather diffused swelling, about the size of a pigeon's egg, over the superior part of the thyroid cartilage, with an increase of the dyspnœa and dysphagia. Some temporary benefit was derived from a liniment; but the symptoms again exasperating, continued to augment till the month of August. During this period, the patient constantly referred the seat of her difficult breathing to the upper part of the larynx. Her cough, at first dry, harsh, and croupy, became latterly attended with a viscid expectoration, tinged with blood. On the 15th of August, Dr. Hall ordered five grains of the quicksilver pill, night and morning; half an ounce of the sulphas magnesiæ twice a week; four leeches to the larynx every other day; and the parts to be kept wet with a lotion, when the leech bites were not bleeding. 22d *of August.* There being no amendment, the mercury was given thrice a day. On the 24th, she was seized with an alarming fit of dyspnœa, with great anxiety of countenance, &c. but it gradually subsided to her usual state.

In consultation with Mr Oldknow, a skilful surgeon of Nottingham, laryngotomy was determined on, and performed by Mr. O. in the following manner. An incision being carried from a little above the thyroid to below the cricoid cartilage, the external surfaces of these were laid bare, partly by the knife, partly by the fingers. An opening was then made between the two cartilages; but this not being sufficient for respiration, a *crucial* incision was made in the membrane connecting the two cartilages. The result being still unsatisfactory, a circular portion, about one eighth of an inch in diameter, was removed from the lower and lateral part of the thyroid cartilage, when the respiration became free, and the patient was relieved. The cut edges of the integuments were kept asunder by straps of adhesive plaster.

In this operation there was some difficulty experienced from the inability of the patient's reclining backwards her head. The larynx could not, in consequence, be brought sufficiently forwards, " and the depth of the incision was necessarily greater than was contemplated." In some judicious observations on this operation, Mr. Oldknow appears to think that an opening between the thyroid and cricoid cartilages possesses advantages over one made lower down in the trachea, " especially when we consider the impossibility frequently experienced of employing a tube" for a permanent opening. " In the present case, the introduction of a probe, merely, induced the most distressing fits of convulsive coughing." The case which we have alluded to above, shows the possibility of keeping a tube permanently in the trachea; whereas there seems little or no chance of the same being possible in the larynx, from the greater sensibility of the parts. We have repeatedly passed large bougies down the trachea through the tube in Mr. Price's case, and on no occasion could he ever feel the bougie. It would sometimes, indeed, set him coughing, from the *organic* sensibility of the mucous membrane; but he never could tell why he coughed, as the membrane possessed nothing of that kind of sensibility residing in the nerves of *touch.*

We sometimes endeavoured to pass the bougie *upwards* through the larynx, to explore the cause of the obstruction; but this always brought on violent *sensible* irritation, and long paroxysms of coughing. Where, therefore, there is probability that a *tube* may be necessary at all, we think the trachea the preferable place for the operation, and that as low down as possible, consistent with safety.

M. Jourdan, in commenting on the preference which Chopart and Desault gave to laryngotomy over tracheotomy, comes to the same conclusion which we have done. " Mais il est difficile, says he, de concevoir quels avantages elle (laryngotomie) peut avoir sur la tracheotomie. Si d'ailleurs on considere que le sommet de la trachée en est toujours la partie la plus sensible et la plus irritable, on se décidera sans peine a la reserver exclusivement pour les cas ou des circonstances qu'il est impossible de prevoir d'avance, empêcheraient de pratiquer la tracheotomie." *Dict. des Sciences Med. Vol.* xxvii, *p.* 273.

Mrs. H. after this operation slept soundly the ensuing night; but deglutition continued difficult, and always induced conghiug during the five or six succeeding days. The voice, of course, was lost. On the day after the operation, mercurial ointment was substituted for the blue pill; and on the 28th, the mouth became sore. Soon after this, Mrs. H. experienced a mitigation of the dysphagia; and, on applying the finger to the wound, she found that the difficul'y of respiration was also diminished.

This amendment continued progressive, and on the 11th of September, the orifice closed, the respiration being free, and the swallowing easy. A few weeks after this, she imprudently exposed herself to cold, which was succeeded by a difficulty of breathing and swallowing, together with a loss of voice. She came once more to Nottingham; was put on the mercurial treatment, " and in proportion as the remedy induced ptyalism, the dysphagia disappeared entirely, and the voice became again improved." The medical and surgical measures pursued in this case reflect great credit on the practitioners engaged therein; and we cannot terminate this article better than in the words of Dr. Hall.

" It may, I think, be fairly concluded, that by means of laryngotomy and of mercury, this poor sufferer was saved from an inevitable death. This case affords, therefore, a striking instance of the efficacy of *modern Medicine and Surgery*; for it is but justice to state, that the means employed were adopted principally on the suggestions of living authors, and especially of those whose names have been already mentioned."*

VII. *Bronchocele.*† We had drawn up an analysis of this pa-

---

* Baillie, Farre, Lawrence, Charles Bell, &c.

† Memoir on a new Mode of Treating Bronchocele. By Dr. QUADRI, of Naples. Medico-Chirurgical Transactions, Vol. x.

per; but just as it was going to press, we received intelligence from a friend at Naples, that the practice of Quadri was found totally unsuccessful, and had been entirely given up. We observe that this is corroborated by Dr. Clark of Rome, in his work reviewed in our present number. We feel it our duty, therefore, to suppress the analysis and lose our labour, rather than contribute to the propagation of an unsuccessful experiment, and uselessly usurp space that may be better employed.

---

### § III. THORAX.

VIII. *Obscure Thoracic Disease.** The difficulty which attends the diagnosis of thoracic affections, especially where the heart is, or appears to be concerned, is well known to practical physicians; and on this account, we shall introduce a brief abstract of the following embarrassing case.

A female child, six or seven years of age, had enjoyed, in the early part of her infancy, a good state of health, and went through the usual disorders of children. A few months previous to the commencement of the present complaint, her appetite failed, her spirits were depressed, and her disposition melancholy. Emaciation and debility succeeded, with the development of an external swelling on the left side of the chest. She was now examined by the narrator, Dr. Kohler of Berlin. On the left side, near to the sternum, about the region of the fourth, fifth, and sixth ribs, there was a preternatural protuberance of the thoracic parietes, with a bluish colour of the skin. Strong pressure on the part produced darting pains. "A throbbing of the heart was clearly evident externally; and on the application of the hand, the violent action of that organ became more forcibly distinguished." The pulse was quick, full, and regular; but fuller and harder in the left than in the right arm. The cheeks were suffused; but the father asserted that when she fell into a passion, which she often did, the cheeks and lips became remarkably pallid. She complained of oppression in breathing; darting pains in the chest, especially in the region of the swelling, increased by cough, which she had together with the throbbing of the heart. She had also pains in the head; flushes of heat about the body; disturbed sleep, in which she often cried out for help, during frightful dreams; fæces and urine natural. To the foregoing symptoms were added the usual indications of worms, and a scrofulous diathesis. The father recollected that she had had a fall on the chest, a few years previously, but without any apparent consequences.

Dr. K. considered that the pain, the cough, and the inordinate force of the circulation, evidently evinced inflammatory action somewhere, which ought to be combatted. He could not decide

---

* Translated from Hufeland's Journal, by the Editor of the Medical and Physical Journal.

whether the function of the heart was disordered from organic disease of itself, or inflammatory lesion of some other part within the chest. The prognosis, however, was unfavourable. He decided first on sedatives and depletion, to combat the inflammatory state of the system; and, secondly, on mild restoratives to support the strength.

In a short time she appeared somewhat improved; anthelmintic medicines were next administered, which brought away some ascarides; but, during the succeeding three months "the thoracic disease varied but little;" at the end of which period, a scrofulous enlargement of the axillary glands appeared, with catarrhal cough, hardness and distention of the upper part of the abdomen, violent pains in the belly, and "more evident swelling of the subclavicular region." The antiphlogistic and vermifuge treatment, which had hitherto been employed, was changed for some light tonic medicines. In about three weeks the patient "was evidently and surprisingly better." Same measures to be continued. In six days more, "symptoms of dropsy first made their appearance"—"the child sleeps almost throughout the day, and is delirious when asleep." In short, to make an end of an almost endless German detail, a colliquative diarrhœa soon terminated the little patient's sufferings. Throughout this diffuse case, little specific information is given respecting the local and interesting phenomenon in the left side of the thorax, with which it commenced; even in the dissection, the chest is slurred over in a few lines, while all the other parts of the body, not at all engaged in the disease, are dwelt upon with true German minuteness. The following are the only parts of the dissection worth record.

The cartilaginous extremities of the first four ribs, on both sides, about their union with the sternum, were wholly changed into a caseous pulpy mass: the sternum itself, carious on the inner surface of its upper part. The lungs nearly sound, though of a dull whitish-yellow colour externally; the left lung adherent to the "diaphragm and intercostal muscles," [costal pleura]. Half an ounce of a turbid yellow serum in the pericardium; heart itself loose and flaccid; the substance of the left ventricle thinner than in healthy proportion to that of the right; foramen ovale open. The liver extended into both hypochondria; the pyloric orifice of the stomach "in a morbid state, as well as the duodenum, which felt thicker than natural." The mesenteric glands much enlarged, some of them the size of an egg. Not a word is said about the tumour which formed the prominent feature of the complaint at first. It is evident, however, that structural disease of the heart formed no part of the complaint originally, or indeed at any period, although the symptoms, at the beginning, were strongly presumptive of such an affection. In this point of view, the case is interesting.

### § IV. ABDOMEN.

IX. *Ileus.** That the establishment of truth, and the advancement of medical science, are the paramount objects of Dr. Abercrombie, we sincerely believe. That this able physician and surgeon will give us credit for similar feelings and impulses, we have no reason to doubt. Under this impression, we shall investigate the subject of the paper in question, with a degree of attention proportioned to the importance of the disease, and the respect which we entertain for the author. If we combat Dr. Abercrombie's theories, it is not with the intention of depreciating his facts; if we endeavour to develope a sounder pathology of the disease, it is for the sole purpose of co-operating with the author in establishing a rational mode of treatment.

We conceive that the pathognomonic or essential character of ileus, is an *inverted motion or action of the intestinal canal*—in other words, that the simplest form in which the disease can exist, (and unfortunately the rarest) is this inversion, unaccompanied by any obstruction in the intestinal tube. Sennertus relates a case of this kind. "*Observavi adhuc aliud ilei et motus intestinorum inversi genus, neque inflammatione, neque ulli causarum enumeratarum, in quodam viro hypochondriaco; in eo enim ita humores sursum ex hypochondriis vergebant, &c. — ut clysterem vomitu rejiceret.*" The following case, from Barthez, is also in point. "A man became afflicted with pains in the stomach and bowels, which daily increased in violence, till they at length deprived him of rest. One day, after a very slight repast, he was seized with a painful spasm in the epigastrium, which was quickly followed by vomiting and convulsive efforts. *Lavements being exhibited, he threw them repeatedly up by the mouth.*" He was cured by antispasmodics, leeches, blisters, &c. Morgagni, in referring to the histories of such cases, says, "you will find among the several observations, that the glysters were thrown up by the mouth, wholly, entirely, purely as they had been applied, nothing at all changed, after they had been in the intestines for an hour, a quarter of an hour, and sometimes only for a few moments after they were exhibited." xxxiv *Epistle*

Not to multiply authorities on this point, the fact is authenticated by Van Swieten, De Haen, and numerous authors of undoubted veracity.† Suppositories even have been rejected by the mouth; a curious case of which is related by Mathieu de Gradibus. "A girl, 12 years of age, was attacked with ileus. The constipation was obstinate, and she threw up fecal matters and glysters entire [ello

* Researches on the Pathology ofthe Intestinal Canal. By John Abercrombie, M. D. Ed. Journal, January, 1820.

† Among others, Sydenham, no mean authority, as a faithful observer of Nature, observes, "quæcunque in intestinis continentur, non versus alvum, sed ventriculum protruduntur, et impetu facto, ad os regurgitant, adeo ut *enemata* quantumcumque aeria, *emetica* evadunt." Chap. iv.

rejeta par le vomissement les matieres fæcales et des lavemens entiers.] These symptoms continuing three days, a long suppository was introduced per anum. It was quickly drawn up into the intestines, and rejected by vomiting." "Aussitot il remonta dans les intestins, et fut rejeté par les vomissemens."*

The above, we should hope, are sufficient to establish the point, that obstruction is not essential to the production of simple ileus; else how could glysters and suppositories pass rapidly from the rectum to the mouth, even in defiance of the valve of the colon.

But how, and why, is this inverted or anti-peristaltic motion produced? Not surely in the way Dr. Abercrombie has pointed out — namely from a portion of intestine being "distended beyond its power of contraction, or paralysed from over-distention;" and that too, by the increased impulse of matters from *above*.† In short, the facts brought forward here, are fatal, at once, to the Doctor's hypothesis, as far as regards what we term *simple ileus*, in which his supposed obstruction from over-distention, accumulation, and paralysis cannot exist. Let us endeavour to account for this inversion on other principles. We ourselves have seen some instances of the simple ileus, and our reflections on the subject had long ago led us to attribute the anti-peristaltic motion to some morbid irritations of the *nervous system*, which play a greater part in pathology than some modern physicians are inclined to allow. This opinion was not a little strengthened by a perusal of the following facts. "Schwartz, says Barthez, has proved by direct experiments, that this inversion takes place and produces vomiting, if we prick various parts of the brain, the fifth pair of nerves near their origin, or the mesenteric plexuses; and Brunner, by irritating the intestiues of divers animals, has excited in these canals convulsive motions, which caused their excrementitious contents to retrograde into the stomach, whence they were discharged by vomiting." "Barthez rapelle que Schwartz a prouvé, par des experiences directes, que cette inversion peut avoir lieu et produire le vomissement, lorsqu'on pique divers endroits du cerveau et du cervelet, ou les nerfs dits de la cinquième paire pres de leur origine, ou les plexus mésenteriques; et que Brunner, en irritant les intestins, meme dans divers animaux, y a excité des convulsions qui ont fait remonter les matières excrementitielles dans l'estomac, d'ou elles ont été chassées par le vomissement."‡

---

* See the account of this case also, in the 3d Book and 14th Section of Bonetus, where he relates some instances himself of a similar tendency.

† It has been said that there is nothing *new* under the sun. Notwithstanding this dogma, we were inclined to give Dr. Abercrombie full credit for originality in this doctrine, till we read in the 34th Letter, and 30th Article of Morgagni, of "those cases in which ileus has happened on account of the *expulsive* faculty being abolished, or from a *loss of tone* in the intestines, according to the opinion of Salius and Ruysch." We cannot say that this accession of authority, however, has occasioned any change of sentiment in our minds.

‡ Monfalcon sur l'Ileus.

The more scrupulously we examine the phenomena of this simple form of ileus, the more we will be convinced that these inverted actions result from irritation applied either to the origins or extremities of the nerves supplying the intestinal canal. Sydenham, whose practice was bad in ileus, entertains some just notions respecting its etiology. "Hujus inversionis (says he) unde dolor exoritur, *duplicem causam*, adsignare licet. Obstructionem, nempe, vel *irritationem*." Chap. iv.

The next least complex form of ileus, is that in which there is a spasmodic constriction, and consequently obstruction, more or less fixed, in one or more points of the intestinal canal, most commonly in the ileum, without inflammation, or any change of structure.

Dr. Abercrombie doubts whether the muscular fibres of the stomach or bowels are capable of being affected with spasm to a morbid degree. But really we see nothing in his arguments to justify this extraordinary conclusion. He first tells us, page 2, that the peristaltic action of the intestines has nothing analogous in any other part of the body; but at page 6, he seems to forget this, for he there tells us, that "our pathology of such muscles must be derived chiefly from the urinary bladder." This is strange reasoning! But first let us ask—What is this spasm which is denied to the intestinal canal? We believe that our best physiologists consider spasm as nothing more than a morbid, and generally a painful extreme of contraction; the opposite extreme of which is paralytic relaxation. Dr. Abercrombie admits the *latter* extreme, but denies the *former*. We shall, at present, avoid the fallacious ground of analogy, and beg leave to take Dr. Abercrombie on the more solid basis of *the symptoms during life, and the appearances on dissection.** No man who has seen a case of ileus, or even common colic, will deny that the symptoms of spasmodic pains are infinitely more prominent than those which might be supposed to attend a paralytic relaxation of the intestines. Even if the pain in ileus were, in a great measure, owing to the unnatural distention, by flatus or other matters, of some part of the bowel, still we contend that this distention would not exist, were it not for a constriction some where else. But the patient dies. We find one portion of ileum unnaturally distended, and another contracted, so as to resemble a cord. We would ask every unprejudiced observer, whether we have not as good reason to con-

---

* Dr. A. instances the urinary bladder, when "distended beyond its power of contraction," as illustrating his doctrine of distention as the cause of ileus. But he takes no notice of the usual *causes* of this vesical distention; namely, spasm, inflammation, strictures, or other organic affections of the urethra or neck of the bladder, or too long voluntary retention of the urine. This part of the analogy very beautifully illustrates the pathology of ileus; but this part is entirely overlooked by Dr. Abercrombie! Dr. A. questions the existence of spasm in the bladder. As a practical surgeon, he must have seen spasmodic contractions of the bladder, from the irritation of calculi, &c. which sometimes become encysted in this way.

sider the *latter* portion of bowel in a morbid state of contraction, as the *former* in a morbid or paralytic state of dilatation? If this contraction be not always very firm, in the dead body, will any candid man assert, on this account, that no spasmodic constriction existed during life?* But the fact is, this morbid contraction is so very generally to be seen, in fatal ileus, where no other visible cause is found, that it has given the name of *Chordapsus* to the disease, from the very appearance described by Dr. Abercrombie, [" a solid cord, white and corrugated,"] as constituting the natural and *healthy* state of the intestine. " Nearly the whole track of intestine (says he) may occasionally be seen in this state in the bodies of infants, who, before death, had been *much purged*, or who had been affected with *diarrhœa*, without disease of the coats of the intestine." Dr. A. does not consider that the irritation of purgatives, or the irritable and sub-inflamed state of the mucous membrane, in diarrhœa, has any share in producing this *chordapsus* of the intestine. He considers the muscular fibres in this greatest degree of *contraction*, as being probably, in the greatest degree of *rest!* Upon such principles, it is needless for us to attempt to reason. Neither shall we vindicate the *supposed erroneous* doctrine that spasm can exist in the stomach, against so inconclusive an argument as Dr. Abercrombie has brought forward against it, namely, that " we find persons labouring under the affection which has received this name, swallowing hot water and other liquids in large quantities." p. 6. We would only just venture to hint, from some little observation and personal sufferings, that *partial* and very painful spasmodic contractions may obtain in the stomach, as well as in other parts of the intestinal canal, without such an obliteration of its cavity as would prevent a man from swallowing hot water and other liquids. But to return to the direct path of investigation. The natural and healthy motions of the intestinal tube, are not only continually varying the calibre of its different parts, but contorting the convolutions of the small intestines themselves, so as to be every moment forming twisted strictures, and perhaps even slight intus-susceptions, that are as quickly unravelled by the next vermicular movement. But let a morbid irritation be propagated to, or generated in this writhing tube, and

---

* Dr. A. refers to the urinary organs for analogies; he cannot, therefore, refuse the following: " This contraction and relaxation (says Sir Everard Home) form the natural and healthy actions of the urethra; but as this membrane, like every other muscular structure, is liable to a *spasmodic* action, which produces a degree of contraction beyond the natural, in which state the canal loses the power of relaxing, until the spasm is removed. When this happens, it constitutes a disease; and is termed a spasmodic stricture. While a stricture is in this stage, it is only a wrong action of the membrane of the urethra; and if the parts are examined in their relaxed state, there would be *no appearance of disease*." If this be true of the urethra, where muscular fibres can hardly be demonstrated, how much more applicable is it to the intestines? Spasm then *may* be relaxed by death, and leave no trace of its existence.

then we shall have *spasmodic* contractions, instead of natural ones, attended with colicy pain, intermitting or remitting; if the spasms intermit or remit, nausea, vomiting, and all the phenomena of ileus. This spasmodic constriction may supervene on simple ileus, (the anti-peristaltic motion of the bowel;) or it may produce ileus, and in either case, we have a combination, more formidable than the simple inverted action of the intestines.

In this kind of constriction, the anti-peristaltic motion is not constantly going on. On the contrary, it appears to take place only when the natural action of the bowels has distended the parts above the obstruction to a certain extent, with the matters contained therein, when the inverted motion really appears an effort of nature to void upwards, what cannot be passed downwards. That ileus, so little complicated as in this form, often destroys life, we very much doubt. Dr. Abercrombie's first case is related as an example;* but it is not very circumstantial; and, at all events, we know that so slight a degree of inflammation in the bowels, as is scarcely cognizable on dissection, will sometimes destroy life. In Dr. A's case, the bleeding and other antiphlogistic measures, too, might have prevented the traces of inflammation being very evident. Dr. A. indeed, acknowledges that this is "an unusual termination of the disease in adults;" but believes that infants are frequently cut off in this manner, by the mere interruption of the healthy action of the intestinal canal.

The *third* form, or rather complication of ileus, which we would advert to, is that in which inflammation is added to spasmodic constriction and anti-peristaltic motion. This is by far the most frequent form which we meet in practice, though the *second* form probably precedes it, in most instances, before we see the patient. Between violent spasm and inflammation there is a very thin partition, which every practitioner ought to bear in mind. We believe that ileus rarely proves fatal without inflammation, when, in fact, we have enteritis to contend with, in addition to the original disease. The following passage is well worthy of quotation; and we would particularly solicit Dr. Abercrombie's attention to it. "Quippe cum plures ab ileo defunctos aperuerim, intestini cujusdam inflammationem, ac sphacelum fere in omnibus, et morbi et mortis causam fuisse deprehendi, *neque mirum hoc, quoniam in membrana tenera et maxime sensili magna solutio continui spasmos et corrugationes dolorificas ita continuas et atroces ciebat, propterea ut* intestini affecti motus peristalticus, quo fæces alvi versus anum protruduntur inhibeatur ac prorsus invertatur." *Sepulchret. Bonet. lib. iii. sect. xiv. observat. xix.*

We do not believe that a sounder piece of pathology, in respect to ileus, has been broached since the days of Bonetus, or one which is in stricter unison with the best principles of practice, as we shall see farther on. The writings of Morgagni, Lieutaud, Bonetus, and all pathologists are full of histories of this form of ileus; and they all concur in shewing not only obstruction but inflammation in the intestinal canal. We shall not, therefore, take up space in relating

* Edinburgh Journal, page 8.

individual cases, but reserve it for more useful and general observations. We cannot, however, pass unnoticed some specimens of what Dr. Abercrombie calls ileus without obstruction; because we think that they are not only misnomered, but misplaced, as bases for a false hypothesis.

In the seventh case, page 13, the lady "was affected with vomiting and pain over the whole abdomen, which was rather tense upon pressure; pulse rather frequent. She was bled, and blistered, and took laxative medicine, which operated freely." Surely Dr. Abercrombie is not in earnest, when he adduces the above as a specimen of ileus. Is the simple act of *vomiting*, a proof of the peristaltic action of the *intestines* being inverted? And are pain and tension of the whole abdomen the symptoms of a local paralytic distention, on his own principles? The fact is, that this patient had a mass of hardened fæces in the colon, which was producing general abdominal irritation, threatening inflammation, with sickness at stomach of course. After bleeding, blistering, and subsequently wine, in considerable quantities, these hardened fæces were brought off "in most extraordinary masses," by injections and castor oil, when she was immediately made well. Where was the ileus in this case? Dr. A. says it was a case in which there was no obstruction. We cannot agree with him. The first purgatives only brought off "thin fæces," which did not relieve the patient. The masses of hardened fæces must necessarily have produced obstruction, though not a *total* stoppage of the passage. Had the latter taken place, real ileus would, in all probability, have supervened.

"Case 8th. A gentleman, aged about 40, (10th November) was seized with vomiting and pain of the left side of the abdomen, his pulse varying from 40 to 60; *took purgative medicine, which operated fully;* and on the 11th, the vomiting had subsided, but the pain continued severe, and was *more general over the abdomen*; pulse 70.—12th. A tympanitic swelling appeared on the left side of the abdomen, which, on the 13th, had extended also to the right side; violent pain continued; pulse natural.—On the 13th, he took purgative medicine, *which operated fully four or five times.*—On the 14th, he was free from pain; but the swelling had extended over the whole abdomen; pulse still natural. On the 15th, the *pain returned with great violence, with vomiting, and frequent pulse.* It continued through the day and night, and he died early on the morning of the 16th. The body was not examined."

As there was no dissection, we will not take upon us to say positively what the disease *was*, (though few will hesitate to pronounce it peritoneal inflammation with tympanites) but we affirm that it *was not* ileus. There was no evidence of inverted motion of the *bowels*, for stercoraceous vomitings are not even hinted at; and from the 11th to the 15th, there was not even inverted action of the *stomach*. There is no evidence of obstruction in the gut, for purgative medicine "operated fully four or five times." If the tympanitic distention then was within the bowels, it must have been gaseous; and what prevented it from passing off, if, as Dr. A. acknowledges, there was no obstruction?

We may remark, "*en passant*," that this patient could not have been Dr. Abercrombie's, for the treatment was exceedingly inert. He, therefore, probably had but an imperfect account of the case.

Dr. Abercrombie's next case, [Case 9, p. 13] has still less pretensions to the name of ileus. There was no vomiting or antiperistaltic motion of the bowels—no accumulation—no obstruction. The case was clearly spasmodic colic ending, as it often does, in inflammation and death. On the 11th of June, 1818, a gentleman was attacked with "violent pain across the lower part of the abdomen, which was *drawn up into balls*." These attacks, he used to have periodically, sometimes returning every evening, for a week together. He was relieved, and his bowels evacuated by an *opiate and purgative*. Two days afterwards, the pain returned with violence, and was not relieved by an opiate. He was not bled till the next morning, by which time spasm had ended in inflammation and gangrene. On many parts of the ileum there were inflamed portions, with effusion of coagulable lymph, and others of a dark colour, approaching to gangrene. An old adhesion between the caput cæcum and ascending colon, evinced that in former attacks inflammation had not been wanting occasionally, though never to a fatal extent. There is not the least shadow of reason for placing this case under the head of ileus.

It is to be remembered that as, in this third form of ileus which we have been examining, inflammation supervenes on spasm and antiperistaltic motion of the intestines; so, on the other hand, the antiperistaltic motion itself, or ileus, very often supervenes on inflammation, and thus becomes a secondary phenomenon attending enteritis. This appears to have led Dr. Abercrombie astray; for he has evidently confounded ileus, enteritis, and spasmodic colic, all under one denomination.

The fourth and last form or complication of ileus, is that in which there is organic or mechanical strangulation of the intestine, as hernia, intus-susception, volvulus, foreign bodies in the tube, and various other causes of obstruction. The histories of such cases are endless. It is proper to state, that in *these* instances, the symptoms are the same as in all the other complications of ileus. The distention, too, of the bowel above the *organic* stricture, is the same as above the *spasmodic* contraction; a plain proof that the said distention was caused by, and did not cause the contraction. We shall make a few observations on some of the principal organic or mechanical obstructions, which supervene on or produce ileus, passing over strangulated herniæ, as too familiar to every reader.

1. *Intus-susception.* This, as we before hinted, probably takes place, in a slight degree, during healthy peristaltic action; but the succeeding vermicular movement undoes what the preceding had done. In severe colics, or where there is much nervous irritation, however, the intus-susception may become fixed, an obstruction produced, adhesive inflammation kindled, and death ensue. In this way, we can see that it may either supervene on ileus, during the inverted action of the bowels in that complaint, or it may precede ileus, and produce it. Peyer, in a great number of experiments, demonstrated the possibility of inducing intus-susception, by irritating the intestines of animals; and, in all human probability, it is in this way, namely, by irritation, that the accident takes place so often in children, whose bowels so generally contain irritating substances and secretions, and whose nervous systems are so susceptible of various morbid sympathies. There is reason to believe, from many cases on record, that these invaginations are sometimes caused by worms. We shall only introduce one instance as an example. M. Raisin relates the case of a soldier, who, in the period of convalescence from a fever, was seized one night with inexpressible anxiety, and unconquerable restlessness. Presently he raised himself up in his bed, gave vent to some groans, and fell down dead. On dissection, the epiploon was found slightly inflamed, as were the intestines, which were amazingly distended with gas. An intus-susception, of about four inches in extent, was found at the superior part of the ileum, above and below which were seen a number of lumbrici.

Although there is no diagnostic symptom by which we can ascertain intus-susception with any degree of precision, yet Montfalcon thinks that, "the violence and fixed seat of the pain, the obstinacy of the constipation and vomiting, together with the rapid prostration of the patient's strength, are symptoms strongly indicative of intus-susception." Even in this dangerous situation, Nature sometimes delivers the patient, when Art is completely unavailing. Hévin has collected many authentic examples of mortified intestine being thrown off per anum. In one case, *(Observations de Sobaux)* twenty-three inches of colon, with the part of mesocolon to which it was attached, were expelled by stool; in another, *(Observations de Salquer)* twenty-eight inches of ileum, in a state of gangrene, were thrown off; in a third, *(Observation de Fauchon)* the cæcum with six inches of colon, and the same of ileum, were discharged per rectum, and in all, the patients perfectly recovered. Dr. Baillie has also recorded a similar event, but we believe the patient died about three weeks afterwards.

2. *Volvulus — Entortillement — Entérélésie.* It is by no means uncommon to find some part of the small intestines so twisted, interlaced, or, as it were, involved in a knot, as to produce complete strangulation, with or without adhesion, inflammation, or gangrene, according to the length of time which the patient had been able to bear up against the complaint. We shall illustrate these by a few examples.

"A youth of 17 years, vomited stercoraceous matter during fourteen days before his death, without passing any thing by stool the while. When the abdomen was opened, a great quantity of gas rushed out. The intestines were vastly distended; some of them as thick as a person's thigh, in certain places." "Aliis vero locis adeo convoluta, intorta et implexa, ut nulla illic nec excrementis nec flatibus deorsum pateret via: sed et vermibus vivis oblongis, quamplurimis repleta erant qui rursum aliis minoribus referti, inequales illos motus efficerant." *Platerus, Obs. Lib.* 3. "Testisque mihi fuit TILEMANNUS, se vidisse aliquando in Sectione Anatomica ab ileo mortui, intestinum ileum contortuplicatum, uti pannus à lotricibus convolutus." *Dietericus Iatrei.*

The following most interesting case we shall translate from Riverius.

"*January* 29*th*, 1644. D. Patris, began to complain of violent colicky pain, occasioned, as was supposed, by exposure to intense cold. The family apothecary threw up a glyster, which brought away much fæces. The pain continuing, two or three other glysters were exhibited, but returned unaccompanied by stools, the violence of the pain still increasing. The next day, Riverius was called in, and prescribed anodyne fomentations and glysters. There was now neither pain nor fever; but the patient vomited at intervals, and no passage could be procured by stool. The disease in a consultation, was considered to be ileus. On the third day, there was fever, with a dry tongue; but there being no pain, it was thought there could not be inflammation of the bowels. The cause was thought to be accumulated fæces, or a contortion of some part of the intestine. Bleeding was thrice performed, glysters were thrown up, and various remedies were tried; but the vomiting and constipation continued till the seventh day, stercoraceous matter being thrown up by the mouth. On the evening of the seventh, the bowels were opened, great quantities of fæces passed by stool, and the vomiting stopped. A diarrhœa continued for five days more, which becoming colliquative, the patient was carried off on the thirteenth day of his illness."

What were the appearances post mortem here? Had no dissection taken place, it would be confidently asserted, that there was no organic obstruction, as the open state of the bowels seemed to prove. Let us see. On opening the abdomen, "inventum est intestinum ileon *tribus complicationibus convolutum*, et quasi compactum in unam massam, circa finem illius. Tota autem illa intestini portio com plicata, gangrena affecta erat, cum portione mesenterii sibi adjuncta." The rest of the intestines were greatly distended. The ileum, above the obstruction, had burst, "à pondere materiæ contentæ in superioribus intestinis," and the excrementitious matters were extravasated into the abdomen. These last had burst into the rectum, and were discharged by stool for several days; consequently, as Riverius justly obsserves, "obstructionis intestinorum apertionem *mentiebantur*: — unde patet alvi fluxum, ileo superveniente, posse interdum medico imponere." *Centaur.* 3. *Observat.* 26.

This case shews us how difficult it is to ascertain the period of transition from spasmodic or any other obstruction to inflammation, and how dangerous it is to keep any other hypothesis in view than the possibility of inflammation.

The appendix ceci vermiformis has been known to occasion fatal ileus by strangling a contigious portion of intestine. Vermiform appendices of this kind are sometimes found on the small intestines, and two instances of their causing ileus now lie before us. The one happened at Lyons, and is stated by Dr. Martin of the Hotel Dieu there. A young man entered the hospital with pain and tension of the abdomen, which took place immediately after lifting a heavy burthen the evening before. He felt, at the time, something crack in the abdomen. All the symptoms of ileus quickly supervening, he died on the sixth day. On opening the abdomen, the intestines were found greatly distended with gas, and the major part of the ileum sphacelated. A vermiform appendix, very similar to that of the cæcum, taking its origin from the ileum, had its other extremity adherent to a portion of neighbouring mesentery, and thus formed an arch over three knuckles of intestine, which were completely strangulated thereby.

The following case, by Renault, evinces the rapid march of inflammation in these mechanical obstructions, which exhibit the same symptoms during life, and the same appearances on dissection, that the spasmodic constrictions do—a convincing proof, that it is obstruction, not paralysis, which is the fundamental cause in both species. " A man felt pain in his bowels, which became greatly aggravated in the night, and next day was succeeded by all the symptoms of iliac passion. He died on the second day of his illness. The abdomen was prodigiously distended; and, on being opened, a great quantity of fœtid gas rushed forth. There was considerable effusion of dark coloured serum into the abdominal cavity. Various portions of peritoneum and gastric epiploon were gangrened. The small intestines universally inflamed; and, in some places, sphacelated. A vermiform process springing from one of the small intestines, seven inches in length, formed a knot around, and strangled a knuckle of mesentery."*

This case elucidates that tympanitic distention which so generally attends peritoneal inflammation, seemingly occasioned by the extrication of fœtid gas. An ingenious explanation of this phenomenon was read, a few years ago, before the Royal Society, by Dr. Granville, some account of which may be seen in the Journal of the Royal Institution, for July 1818. Dr. Granville, in a late publication,* states, that he has now under his care a case of that rare disease, œdopsophia, or "*flatuum ex utero per vaginam, emissio,*' which he considers as of the same nature as tympanitis. " I am strongly tempted to believe, says this ingenious and erudite physician, that the extricated air in this case is a combination of sulphur and azote, from a decomposition of the albumen of the blood, in consequence of inflammation of the womb. This fact I was the first to notice in a communication read before the Royal Society in May 1818, giv-

---

* *Dict. des Sciences Med.*

† Report of the Practice of Midwifery at the Westminster General Dispensary. By A. B. Granville, M. D. London, 1819.

ing an account of a new compound gas, produced by peritoneal inflammation. In both cases, the albumen is separated from the blood by the process of inflammation, and is ultimately decompoesd by some other process, yet unknown; giving rise to carbonic acid gas on the one hand, and to sulphuretted azote on the other; producing, as in the former case, *œdopsophia*, the disease here mentioned: and, in the second, abdominal tympanitis." *Loco citato.*

We may here just observe, *en passant*, that the distention within the intestines, *found on dissection*, should be regarded with a suspicious eye; and, in our idea, it affords a very unstable basis for an etiological hypothesis. Every one knows what an immense extrication of gaseous fluid takes place in the bowels, immediately the vital principle becomes extinct. And as this is generally the case *in the intestines*, for some time prior to the death of the patient in ileus, so there is every reason to believe that the greater part of the distention, seen in dissection, takes place at a very late period of the disease, or even after death; and so far from being a cause, it is quite a secondary, or ternary effect of the morbid state in ileus.

M. Fages relates an interesting case of fatal ileus, caused by a portion of ileum becoming incarcerated in a sac of peritoneun, situated on the anterior part of the psoas muscle; and many instances are on record where the intestines have been strangulated by bands of the epiploon, and by knuckles of gut passing into rents of the same.

III. *Ileus from Fæces or foreign Substances lodged in, and obstructing the Intestinal Canal.* A girl, seven years old, was afflicted for several days with a diarrhœa, which the old women at length put a stop to by exhibiting large quantities of quince fruit. Very soon after this sudden suppression of the diarrhœa, she was seized with severe and painful tormina of the bowels, together with distention of the abdomen. The physician being called, endeavoured to restore the diarrhœa, but in vain; no stools could be procured; stercoraceous vomiting came on, and death ultimately closed the scene.

*Dissection.* "Aperto corpore, cæcum intestinum coangustatum constrictumque, adeo obhærescente, et interioremque ductum obturante cydoniato, deprehensum est, ut illac nullo modo prorsus quicquam posset pervadere." A little above the obstruction the intestine had given way, and fæces were extravasated in the belly. *Fenelius Pathol. lib. 5. Cap. 9.*

Lamartiniere relates the case of a young gentleman, who, for the purpose of stopping a relaxation in his bowels, indiscreetly eat a great number of hard boiled eggs. Unconquerable constipation followed, with obstinate vomiting, till death put an end to his sufferings. A column of intestine was found firmly impacted with hard substances, above which, the gut was enormously distended.

In the 4th volume of this Journal, (Monthly Series) Mr. Morrison, a navy surgeon, relates the case of two marines, who ate, at one meal, *forty-four* eggs. They both died; one on the second day, with ileus; the other on the eighth, with inflammation of the abdominal viscera, especially the stomach and liver.

Chronic strictures of the colon, and even of the rectum have induced ileus, of which we could adduce several examples. But we think enough has been said.

### TREATMENT OF ILEUS.

We have endeavoured to shew that ileus presents itself, in practice, under four principal forms; viz.

1st. As an inverted or antiperistaltic action of the intestinal canal, without obstruction, spasmodic, inflammatory, or mechanical; dependent, as we imagine, on nervous irritation, general or local. This is the simplest form, and is so rarely met with, that its consideration can have but little influence on our practice. Indeed, we cannot positively decide on its presence, unless we see glysters vomited up; for numerous experiments prove, that if ligatures are made on the lower portion of ileum, the animal will still vomit *stercoraceous* matters, shewing that in such cases these matters acquire a fæcal smell and appearance, without having descended into the large intestines. We have, ourselves, seen two instances, where the glysters were so unequivocally thrown up from the stomach, as to leave no doubts in our mind; and we have proved the existence of this form by unquestionable evidence in the beginning of this paper. The treatment, in cases of this kind, is sufficiently indicated. Opium, united with calomel, is the principal remedy to be depended on; for the disease is, in fact, a cholera, the current of which is turned entirely upwards. The warm bath, opiate glysters, blisters to the abdomen, and blood-letting to guard against inflammation, are also to be had recourse to, if the symptoms do not quickly subside.

2d. In which there is spasmodic constriction, (either as cause or consequence of inverted peristaltic action) and consequently obstruction, more or less fixed, in some part or parts of the intestinal canal, unattended with inflammation or mechanical impediment.

3d. In which inflammation is superadded to the spasmodic constriction.

4th. In which there is mechanical strangulation, with or without inflammation, and whether preceding or supervening on the simple and spasmodic forms of ileus.

Now the practical point is, can these three last forms of ileus be distinguished at the bed-side of sickness? Between the two last, we believe no man, who knows the uncertainty of diagnosis, would venture to lay down a positive distinction, from the symptoms, during life; and between spasm and inflammation, *in the intestines*, there is, as we before remarked, a very thin partition! The diagnosis, indeed, is accurately laid down, *in books*, but we have seen it often lead astray *in practice*.

The symptoms common to all forms of ileus are, pain, more or less violent, and more or less fixed, in some part of the abdomen, generally about the umbilicus; obstinate constipation; vomiting. It is, we believe, by the degree of severity in these, and the *plus et minus,* in the other symptoms which arise, that the best practitioners judge of the nature and danger of the individual case. Thus, the less fixed and the less constant the pain is, so much the more favourable our prognosis. Provided there is a clear intermission, or well marked remission of pain, its great violence, we have generally observed, during the paroxysm, is more indicative of spasm than inflammation or volvulus, and therefore less dangerous, at the beginning; but in violent spasm of the intestines, as we before remarked, inflammation is ever imminent, and ever to be dreaded. The pulse is exceedingly fallacious in all intestinal affections; and the absence of pyrexia, or even pain, *while no stools can be procured,* is never to lull us into a false security.

In common *enteritis,* the pain is more diffused than in ileus; the constipation is less obstinate; the vomiting supervenes on the inflammation; is less violent, and rarely brings up fæcal matters; indeed, it appears to be merely an inverted action of the stomach, from sympathy with the intestines, and is a common phenomenon, or rather epiphenomenon, in peritoneal inflammation also.

If the ileus results from fæcal accumulation, an accurate examination of the abdomen will generally detect it, and the history of the case will lead us to suspect the circumstance. It is almost needless to say, that the groins should always be carefully examined, lest a strangulated hernia be the cause of the symptoms. Monfalcon lays down the following train of phenomena, as indicative of strangulation of the intestinal canal from an internal cause. "Painful tension of the abdomen; obstinate constipation of the bowels, on which the most irritating lavements have no effect; hiccup; nausea; vomiting, at first of half digested aliment, and ultimately of stercoraceous matters; extreme general uneasiness; pulse small and cordy; cold, clammy, and partial sweats; coldness of the face and extremities; great alteration in the countenance; hollowness of the eyes; tumefaction of the abdomen; pain, more or less violent, in some part of the belly. When the respiration becomes feeble, with tendency to somnolency, and a sudden alleviation of the pain, death is at hand." The above symptoms indicate, with sufficient certainty, an obstruction of some portion of the intestine; but the exact nature of this obstruction no man can positively say.

We shall now proceed directly to the treatment. In the simple, but unfortunately not common, form of ileus, consisting of merely an inverted peristaltic action of the intestines, from irritation, and unaccompanied by obstruction, powerful antispasmodics, as opium, æther, and effervescing draughts, with sedative glysters, are evidently indicated; and in the very few instances of simple ileus which we have met, this treatment has been quickly efficacious. But as this form is not only rare, but liable to be confounded with, or quickly terminate in, those forms in which there is obstruction, we dare not safely allow our treatment to proceed upon the purely antispasmodic plan. The very same remark will apply to those forms of the disease in which there is only spasmodic obstruction to all appearance. The transition from spasm to inflammation, volvulus, intus-susception, &c. is often so sudden, and the difficulty of distinguishing between it and them so great, that we should never lose sight of these last, in our treatment.

We believe then, that whenever there is *pain* in any part of the abdomen, we should bleed, both generally and locally, while we exhibit a combination of sedatives and cathartics. Two or more grains of opium, we have usually combined with ten grains of calomel, and as many of compound extract of colocynth, to be taken at once. Large emollient glysters, with laudanum and salts, are to be thrown up, while the warm bath is preparing. Fomentations to the abdomen, after leeches, may precede large blisters, which are useful auxiliaries, though too often neglected.

When Dr. Abercrombie comes to the treatment of ileus, he appears so stricken with the inapplicability of his theoretical principles, that, with an ingenuousness characteristic of great minds, he acknowledges that he " does not presume to think them so established as to be applied to the treatment of ileus."* But surely that theory is exceedingly fallacious which is not drawn *from* the best methodus medendi, as well as from symptoms and dissections. We have always made it a rule to instantly reject the hypothesis which ran counter to any one of the three data above mentioned.

Let us apply Dr. Abercrombie's " remedy of most general utility, the tobacco injection," to his theory of ileus. " The obvious effect of this remedy, says Dr. A. upon the system is to produce *relaxation of all muscular parts*, and the mode of its operation in ileus is involved in considerable obscurity." Why, yes. If Ileus depend on a paralytic or over-distention of a portion of intestine, and this weakened and paralysed part be restored suddenly to healthy action by a remedy which " produces relaxation of *all* muscular parts," then " chaos is come again," and it is in vain to ever reason upon a medical subject in future! But let us apply the *modus operandi* of the tobacco infusion to the simple, and we trust, natural view of the pathology of ileus, which we have taken throughout this paper, and all this obscurity vanishes; the mind finds a resting place amid the wilderness of conjecture, and feels that pleasing sensation which always results from the " rerum cognoscere causas."

We have endeavoured to shew that the *contracted* portion of intestine, however induced, below the distended portion, was the *fons et origo mali*; and what can be more natural than that a medicine which relaxed *all* muscular parts, should operate beneficially here? The thing is so self-evident, that we will not insult the understandings of our intelligent readers, by saying a word in explanation of the principle. Who can fail to perceive that Dr. Abercrombie, in the following passage, by confounding " tonic power" with spasmodic or other morbid contraction of the gut, has led himself into all this labyrinth of obscurity?

---

* Edinburgh Journal, p. 23.

"I have supposed (says he) that, in this disease, the upper part of the canal is healthy, sometimes in strong action; that a part below this is inactive from distention, and that the lower part is *healthy and contracted*, being kept in that contracted state by its *tonic power*, and the suspension of the action by which, in the healthy state of the parts, it would have been distended. A certain force is, indeed, acting upon it by the propulsion of matters from the upper part, but this acts with little effect after being communicated through the intermediate portion, which is in the state of an inanimate canal. It is therefore unable to overcome the *tonic contraction* of the lower part, *which thus opposes an obstacle* to the parts recovering their healthy relations. The same observation applies, if we suppose that the distended part itself retains some degree of action, though feeble and imperfect. Now, in this state of the parts, could the tonic power of the lower part be, for a time, considerably diminished, it might perhaps be brought, as it were, more into unison with the other parts; might be dilated in the natural manner by the weakened force which is acting upon it; and the parts might thus be enabled to recover their healthy relations. Is this the action of the tobacco injection?"

If it be, it is a marvellously accommodating action. It relaxes the fibres of one portion of intestine; (one would almost suppose in compliment to Dr. Abercrombie's hypothesis) but there it suddenly stops, and produces no relaxation in the portion of intestine above this! Thus too, the *constricted* part of intestine had nothing to do with the *pathology* of ileus; it was, in fact, in a state of perfect health and ease. But when we come to the treatment, this comparative exuberance of health stands in our way, and we are forced to apply the infusion of tobacco to dissipate it! In other words, Dr. Abercrombie first laboured to prove that the *contraction* of the gut below, was the *consequence* of "the suspension of action from above," [p. 7] and now he labours to prove that by taking away this *effect* the cause of it will cease! Can Dr. Abercrombie reflect upon these things, and not see, at once, that he has confounded cause and effect, and that he has been led into erroneous reasoning by mistaking morbid constriction for healthy tonicity?

We have paid Dr. Abercrombie a great compliment by investigating so narrowly his doctrines, on this point, which are calculated to lead to the most destructive practice. One of our cotemporaries, (of very superior discernment) enamoured of the paralytic doctrine in ileus, wonders that Dr. Abercrombie did not suggest *wine* or other stimulants as the radical treatment, which would aim, at once, at the origin of the disease!

We agree with our author in his *practical* precepts. He very properly warns us against active purgation, when there is already "a strong, though ineffectual effort to overcome some interruption to the healthy action of the canal." It is on this principle that we always combine a sedative with the cathartic; and that we employ blood-letting, not only as an antiphlogistic, but as an *antispasmodic* remedy of paramonnt importance. Dr. A. observes "that a full dose of opium is sometimes followed by the result which we have sought for in vain from the most powerful purgatives;" a fact, by the bye, which we should conceive is much more in favour of a spasmodic than a paralytic cause of the disease. The following passage respecting tobacco injection, we gladly quote on such respectable authority, though the remedy has been employed for a very long time in ileus.

"It should be begun with caution; perhaps for an adult, in the quantity of fifteen or twenty grains infused in four or six ounces of hot water. After the interval of an hour or two, it may be repeated in a little larger quantity, until such effects are produced by it, viz. slight giddiness and muscular relaxation, as shew that it is exerting its proper effect upon the system. It may then be repeated, at proper intervals, a great many times, if the case do not yield. With these precautions, I have given it in states of great nausea and exhaustion, with the effect of diminishing instead of increasing them; and, in one case, to a child three years of age, with the happiest result. In one of the most severe and obstinate cases I have had occasion to treat, it was repeated nearly twenty times with various partial effects, and at length with complete success."

We have not had occasion to try the effects of cold water externally, or by glyster, as mentioned by Dr. Abercrombie, but we confess we would be more inclined to employ the warm fomentations and enemata.*

In the first number of this Journal, page 133, Dr. Porter, of Bristol, relates an interesting case of ileus, where stercoraceous vomiting had taken place to a great extent, and where five grains of solid opium were exhibited, a large blister applied, and a cold saline enema thrown up. These stopped the vomiting, but did not procure stools. Ten grains of calomel were next ordered, and small doses of the oleum terebinthinæ every hour. On the succeeding day, the fæces appeared in abundance, and recovery was complete.

Sydenham's practice in ileus was as inert as his theory was erroneous; but his Translator, Dr. Swan, makes some very judicious remarks on the treatment of this complaint. "A proper quantity of calomel, (says he) made up into a pill, will more certainly pass than any thing else; and for fear that, in a dose of about twelve grains, it should irritate the stomach too much, it may be given in a less quantity, and repeated as there is occasion; and an opiate may occasionally be mixed with this or other pills. And as the most plentiful bleedings ought to be used, and fomentations frequently applied in this case, there seems to be little danger of inflaming by calomel."

---

* "Home d'Edinbourg, assure que l'ether sulfurique à l'interieur, combiné avec les pediluves *d'eau froide*, lui a parfaitment bien reussi. De Haen dit s'être bien trouvé des lavemens excitans avec *la fumée de tabac*." *Montfalcon.*

There are some instances on record, where spasmodic colic has been resolved by dashing cold water on the lower extremities, or walking on a cold floor.—Rev.

We may here remark that the vomiting in ileus has always appeared to us to be a salutary effort of Nature to remove by the mouth what could not pass downwards, as well as to determine to the skin, and obviate inflammation. On this account, we have never been solicitous to stop the vomiting, except by removing the cause of it. Barthez is in the habit of ordering leeches to the anus, while glysters of herby decoctions, with salts and laudanum, are thrown up into the intestines. He gives assafœtida and camphor, in small but repeated doses, by the mouth. Quarin speaks favourably of a solution of tartrite of antimony in glyster, which acts, probably, on the same principle as the tobacco enema.

Where intus-susception is suspected, it has been suggested to inject large quantities of liquids into the intestines, per anum, with the view of diesentangling the obstruction ;* and Mr. A. Blacklock, of Dumfries, from experiments on the dead body, asserts that air thrown up by a pair of bellows, is exceedingly powerful in removing intus-susceptions. We need hardly remark that the practice of swallowing quicksilver, or leaden bullets, is totally unwarrantable ; and the interesting case of gastrotomy, which we have detailed in the second number of this Series, page 255, affords little prospect of success from a surgical operation in such desperate circumstances. The cases of Mr. Smith, in the ninth vol. of the Ed. Journal, treated partly by *cold water*, do not appear to us at all in point ; and notwithstanding the praises bestowed on this remedy by Alexander of Tralles, Septal, and Hoffman, we should be inclined to believe with Monfalcon, that " La méthode réfrigérante, dans le traitement de l'iléus, a été entierement abandonnée et devoit l'etre."

This article has extended much farther than we expected ; but we hope that we have not taken up our own, or our readers' time unprofitably. If it contain aught of merit, it is attributable to Dr. Abercrombie, whose interesting paper excited our attention to the subject, and whose other papers will call forth similar inquiries. We have examined Dr. A's doctrines with a critical closeness proportioned to the esteem in which we hold him, and the honour which we mean to pay him in our future investigations and strictures. We have plenty of subjects for the exercise of criticism ; but we have no desire to measure our strength with any but the strong.

X. *Peritonitis.*† Acute rheumatism, while it retains its seat in the tissues of the extremities, is a painful, but not a dangerous disease. When a metastasis to an internal structure takes place, the danger is great ; and the patient is never safe till the original affection is restored, or at least till the metastatic action is completely subdued. The serous membranes of the brain, heart, and lungs, are those which generally suffer in translated rheumatism. The abdominal

---

* See Mem. Med. Society of London, vol. ii.

† Case of Acute Rheumatic Inflammation, terminating in Peritonitis. By E. M'Dowel, Surgeon. Dublin Hospital Reports, vol. ii.

viscera are more rarely affected. The following case, however, shows that they are not exempt from these dangerous visitations of a migratory disease.

*Case.* Bridget Daly, ætat. 28, was admitted into hospital on the 22d of May, labouring under acute rheumatism, and complaining of excruciating pain in her knees, which were tumefied, in consequence of effusion into the articulations. The slightest motion was intolerable; she had thirst; was restless and anxious; pulse quick and full; temperature of surface increased; bowels constipated.

This attack commenced about three weeks after parturition, with rigors, heat, and thirst. In a day or two after its commencement, she was affected with general soreness, followed by pain and swelling of the knees. V. S. on the 23d and 24th. Purgatives. Prescribed a pill of calomel, antimony, and opium, 4ta quaque hora.

25th. Fomentations to the knees, and an anodyne draught ordered.—26th. A copious perspiration had not brought relief. To omit the pills, and take decoct. cinchon. with ant. tart. every four hours. Intense pain in the knees, which are more swelled. Œdema of the right leg was observed to day. Twelve leeches applied; tepid bath.—28th. Pains greatly relieved; swelling of the joints diminished; tepid bath repeated at her own desire.

On the 2d of June, an alarming change for the worse. She was found sitting up in bed, leaning forward, seemingly in great agony, the abdomen being very hard, tense, and painful on the slightest touch; pulse quick, small, and very feeble. On inquiry, it was learnt, that on the 27th she had been affected with pain in the bowels and griping, which were removed by a dose of castor oil. She was bled immediately, and eighteen leeches were applied to the abdomen, followed by fomentations and glysters, with small doses of neutral salts. Blood buffed and cupped. The disease seemed to remit in severity, after the bleeding; but the abdomen was still tender. At night the symptoms of peritoneal inflammation recurred, and she sunk in a few hours.

*Dissection.* Abdomen tumid; small intestines much inflated, large intestines contracted. Peritoneum highly inflamed, being uniformly coated with coagulable lymph of a dusky hue. On raising it, the subjacent membrane was found minutely vascular, and of a bright red colour. Considerable effusion of serum in the abdomen, with flakes of lymph floating in it. The peritoneal tissues of the uterus and ovaria, covered with a thick layer of lymph. The *mucous* membrane of the stomach shewed marks of inflammation, but that of the intestines was healthy.

In the knee joints, the whole of the synovial membrane internally was covered with lymph, closely adherent to it; in each articulation about two ounces of fluid, resembling that found in the abdomen. Bands, formed of lymph, stretched across the articulation, in several places. The surrounding fibrous structure was unaffected. The cartilages and bones sound.

We are convinced, from many circumstances which have fallen within the range of our observation and reading, that the warm bath is a dangerous remedial measure in most *acute* diseases; but especially in acute rheumatism. When there is what has been called a determination to any *external* part of the system, the warm bath must have some tendency to increase that determination. But this is not the danger to be apprehended. It is the subsequent *recoil* from the surface to the interior which is to be dreaded, and which may establish a point of irritation and inflammation there, that absorbs the preceding disease, or invites its translation to itself.

XI. *Lithotomy.** The other great operations in surgery have undergone the most important changes in our own times, whilst on lithotomy surgeons have been chiefly occupied in bickerings about the preference of this or that form of instrument for the performance of the operation. An Alanson, an Abernethy, a Cooper, a Lawrence, and a Thomson, have carried the operations of amputation, of aneurism, and of hernia, to a degree of perfection unknown to our predecessors in the healing art; while the operation of lithotomy remains unchanged, after all that has been written on it, since the time of our celebrated Chesselden. On this account we read with unusual interest and pleasure the new method of operating, adopted by the Italian surgeon, which, from its novelty and importance, we think deserving of a full *notice.* The subject of Professor Barbantini's operation was a countryman of about 50 years of age, whose constitution had suffered considerably from the long continued irritation of his distressing disease. On examination, the calculus was discovered to be very large, bearing down by its weight the fundus of the bladder, so as to form a tumour projecting into the rectum, and occupying the space from one tuberosity of the ischium to the other. This calculus being evidently too large for extraction by the lateral operation, and knowing well the difficulty and danger of breaking down the stone in the bladder, Professor B. gave up all idea of it. The high operation still remained; but he considered that method as affording him little hope of success. "L'Esito troppo spesso infelice di questo modo di operare, me lo aveva fatto sempre riguardare come uno di quei mezzi, che l'arte salutare adopera con avveduto consiglio, perchè niun altro ne conosce men arduo, o men pericoloso, e che la necessità sola consiglia." The extraction, by the rectum, was then determined on. Having first introduced a sound into the bladder, he divided the sphincter ani, and integuments of the perineum, to the extent of about half an inch from the anus; introducing his finger into the wound, he now felt the sound, and guiding his

* Account of the Extraction of a large Calculus from the Urinary Bladder through the Rectum. By Dr. N. Barbantini, Professor of Clinical and Operative Surgery in the Royal Lyceum, and Surgeon of the Hospital of Lucca. [ From our Correspondent at Rome. ]

scalpel by the finger, made a small incision into the bladder, cutting upon the sound a few lines beyond the prostate; into this incision he introduced a long director, which he insinuated between the stone and fundus of the bladder, on which it rested, and on it divided that viscus to an extent sufficient to admit the passage of the stone. The parts divided in this operation were a few fibres of the transversus perenei muscle, the whole of the sphincter ani, on its anterior part, the same part of the rectum, and the anterior inferior part of the fundus of the bladder; the quantity of blood lost was very trifling. The extraction, from the great size of the stone, was attended with some difficulty, and required much more force than is necessary on ordinary occasions. The stone, which was something of an oval form, weighed nine and a half ounces; its length (measuring from the engraving now before us) three and a half inches; its greatest breadth two inches and five-eights.

A slight degree of fever occurred on the second day after the operation, but soon disappeared, and no untoward symptom occurred during the progress of the cure. In dressing the wound, the sphincter ani was kept from adhering too quickly, that the fæces might have a free exit, and run no risk of being forced into the bladder during their evacuation. For the first eighteen days the urine passed entirely by the anus, with the exception of a few drops only; which, from time to time, escaped by the urethra. On examining, at the end of this time, the parietes of the bladder were found thickened and hard, and to its internal surface some gravelly particles were found strongly adherent; these were separated by the finger, together with several portions of a new formation of a firm membranous substance, *(pseudo-membrana molto consistente)* and this operation repeated till these substances could no more be felt. An elastic catheter was now introduced through the urethra into the bladder, and by it the urine passed almost entirely; when the fæces were fluid only a small quantity entered the bladder, passing off with the urine by the catheter, and producing no inconvenience. At the end of fifty days from the operation the patient felt so little inconvenience, that he requested to return home. The wound, on examination, was then found diminished to half its original size, the healing process having taken place in the posterior part of the wound. The thickening and induration of the bladder had also nearly disappeared. The catheter was now laid aside, and at the end of thirty days more the patient wrote that he was perfectly well; that he passed his urine at pleasure, and no more passed by the rectum. We have thus given a pretty full account of Professor B's case, which he has related plainly and accurately, because we think it a valuable addition to our stock of surgical knowledge, as shewing (proving) that there is a safer and easier method than any before practised, of extracting large calculi from the bladder. We have even our doubts whether, from the facility with which this operation may be performed, the absence of all risk of hæmorrhage, the ready exit which it admits of to the stone, and consequently the much less hazard that it presents of being followed by the dangerous consequences resulting from laceration of the parts during extraction, it may not, upon trial, be found preferable to the lateral operation in a great majority of cases. The readiness with which the urine flowed by the catheter, when introduced at the end of eighteen days, is very satisfactory, as at that period the wound of the bladder remained of its *original* size; and hence we may conclude, that had the catheter been introduced sooner, the same thing would have happened. We can see no objection to its being introduced from the day of the operation; on the contrary, its being so may facilitate the healing of the wound, by removing the irritation arising from the passage of the urine by it. But our limits forbid us to enter farther on this subject, convinced that it is sufficient to have made our countrymen acquainted with the case, to call their attention to it. With regard to the origin of the recto-vesical operation for the stone, our author observes, that Haller, in his Bibliotheca Chirurgica, mentions an Italian surgeon of the name of Végèce who advised it. *Jubet per vulnus recti intestini et vesicæ, aculeo lapidem ejicere;* and lately, Dr. Sanson (in the Dictionaire des Sciences Medicales, Art. Lithotomie) also recommends it; but it has been first put to the test of experience in that country where the idea of its practicability first originated. Professor Barbantini certainly deserves the highest credit, and the thanks of mankind in general, for shewing a method of relieving them from one of the most painful and harrassing diseases to which they are obnoxious; and if, by thus giving circulation to his case, we shall be the means of relieving a single individual that would otherwise have been carried by his malady to the grave, it will be a cause of infinite gratification to us.*

XII. *Anteversio Uteri.*† In this little volume of Dr. Granville's we have observed a great many interesting cases, and are sorry we cannot devote space for a general review of the work. We shall, from time to time, however, introduce particular cases, under their appropriate heads, which will afford sufficient indication of the merits of the publication.

Every displacement from the natural position of the uterus demands serious attention; and we fear that manual examinations are not so frequently made as they ought to be, from false delicacy on one side, if not on both. Eliz. Stedman, ætat. 28, of very nervous temperament, had aborted three times, at eight and ten weeks. She became weakly in consequence, and very irritable. In this state she had continued several years before she came under the care

---

* The high operation was lately performed in the Santo Spirito Hospital, at Rome, on a very unfavourable subject, with complete success. CORRESPONDENT.

Our Correspondent had a conversation with Mr. John Bell at Rome, relative to the above operation, and informs us that the latter "thinks very well of it." EDIT.

† A Report of the Practice of Midwifery, at the Westminster General Dispensary, during 1818, &c. &c. &c. By AUGUSTUS BOZZI GRANVILLE, M. D. F. R. S. F. L. S. M. R. I. Physician in Ordinary to His Royal Highness the Duke of Clarence; Licentiate of the Royal College of Physicians, &c. Octavo, pp. 220, 1819.

of the Reporter, who found her affected with hysteria, impaired digestion, and suspended menstruation. These complaints were, in a great degree, removed by appropriate remedies; but still she suffered excruciating pains in the back, great heat and irritation in making water, with sense of weight immediately behind the pubis, bearing down, and inability to retain her urine. These symptoms were accompanied by a thin serous discharge, which excoriated the parts. On examination, Dr. Granville found the vagina relaxed; the urinary bladder weighed down, and pressed against the concave surface of the pubis; the os uteri turned upwards and backwards, while its fundus had fallen forward, and rested upon the bladder. Dr. G. endeavoured to rectify this mal-position of the parts manually, and succeeded in giving strength to the parietes of the vagina, and checking the discharges by the cold bath, and the following vaginal injection which the Doctor recommends. ℞. Decoct. quercus ℨx; decoct. ros. caninæ ℥vj; zinci sulph. ℈j; super-sulph. aluminis ℈iß; alcohol. ℈ij; infus. opii aquos. ℨß; m. ft. injectio.

What contributed most to the poor woman's recovery, however, was, "an improved bandage in the shape of stays, by the help of which the abdominal muscles were supported, and all the viscera retained *in situ*. The patient became pregnant.

XIII. *Genital Organs.** As it does not enter into the plan of this, nor indeed of any other Journal, to give regular analyses of elementary works, but only of the improvements which they introduce, or the peculiarities which they may display; and moreover, as it is our intention shortly to revert to the subject of syphilis generally, so we shall be brief in our notice of the volume under review.

Mr. Foot, we believe, is one of the oldest practitioners of this metropolis, and consequently his long experience gives his observations a title to respectful attention. By a chain of historical investigation, he maintains the commonly received opinion, that the syphilitic infection was introduced into this country from America through the medium of Columbus's crew. He also argues, and we confess, with great ingenuity, for the identity of the chancrous and gonorrheal poison; accounting for the dissimilarity of its effects, by the difference of structure in the parts to which the matter is applied. Gonorrhœa Mr. Foot considers to be a specific inflammation of the mucous membrane of the urethra, over which mercury has little or no control. He believes that while the discharge continues, the venereal virus is rarely absorbed into the constitution. Experience, he believes, tells us that those who wash the parts and keep them clean, after connection, are seldom infected. "The best remedy he knows is the act of urining, and washing the prepuce and glans repeatedly well, and wiping them during that act." Next to this is an early injection and lotion of solution of blue vitriol.

* A complete Treatise on the Nature, Symptoms, and Cure of Lues Venerea, Historical, Theoretical, Practical, and Original. A new edition, amended and corrected. By Jesse Foot, Esq. Surgeon. 1 vol. Octavo, pp. 420, London, 1820.

The following injection he has found very useful in the first stage of gonorrhœa. It should be thrown up six or seven times a day.

"Dissolve blue vitriol in a sufficient quantity of spring water, precipitate the solution with a sufficient quantity of lixivium tartari, (which may be known by the effervescence ceasing) suffer it to separate, and pour off the clear liquor; then wash the precipitate with warm water, set it by to subside, decant the clear liquor, and repeat the process with fresh quantities of warm water, till it become insipid and tasteless, at least of the salt; then filter the solution, and reserve the precipitate. Dissolve as much sal. volat. sal. ammon. in distilled water as it will take up, and filter it. Mix as much of the above precipitate with the filtered solution as it will dissolve, which reserve for use."

Five drops of this solution to an ounce of water is the proportion used. When, from neglect, the inflammation has pervaded the whole of the urethral lining, the plan for abating the inflammation must be soothing.

"Constant injections of warm milk and water, and a moderate proportion of mercury should be administered daily. Four grains of the quicksilver pill, with one grain of opium, will not only be necessary to guard the constitution, but will, in process of time, so far affect the secretion of the mucous membrane, as to render it less *exposed* (we should rather say less *sensible*) to the irritability (irritation) of the virus.'" 115.

If the symptoms of inflammation and irritation augment, the discharge becoming thinner and mixed with blood, with deep-seated pain towards the anus, &c. leeches, in addition to the soothing plan, should be applied to the perinæum, and the warm bath or fomentations, with antimonials.

Sometimes the inflammation runs so high that the discharge is altogether checked; and this our author considers the worst condition of gonorrhœa. "The origin of most of the obstructions in the urethra is from this inflamed state of parts." In cases of chordée, hæmorrhage is rather favourable.

"The termination of the inflammatory state may be known by a return of a kindly discharge, which will be ropy and thick, leaving a daub upon the linen like white paint, and which, gradually becoming less and less, will cease altogether. The parts will again enjoy their natural functions, and then the disease will be at an end."

But as this is not always the case, the ulterior consequences of gonorrhœa are to be carefully attended to. Mr. Foot thinks that the virus often lurks under what is termed a gleet.

"If the discharge be supported solely by a continuance of the virus, that will be discovered by the freedom of the stream of urine, by no particular part being affected, by the discharge being regularly distilled, and by the sensation which is felt being generally diffused along the urethra: or it will be discovered, a posteriori, by the discharge kindly declining, through the use of the vitriolic injection, or by the inflammation increasing upon ceasing to inject." 109.

Our author makes many judicious observations on the use and abuse of injections, which deserve the reader's attention. He remarks that, in the cure of gonorrhœa, although cooling laxatives are proper during the inflammatory stage, yet that the "*purgative system*" is a bad one. "There is no method of cure (says he) I have ever seen adopted, which protracts a gonorrhœa so long as this; and there is no method of cure which exposes the patient to an absorption of venereal virus more than this; a swelled testicle often comes on from this cause, a dysury yet oftener; and the discharge, from this treatment, will be spun out to a tedious length. I think, upon the whole, it is safer to do nothing than to attempt a cure by purging medicines." 123.

On the subject of phymosis, Mr. Foot gives many useful hints. Among other measures he advises that "the patient should always urine with the penis in warm water, to wash away the urine confined within the swollen prepuce."

From the circumstance of Mr. Foot's work being principally elementary, we must necessarily pass over the various chapters descriptive of the symptoms, distinctions, &c. of primary and secondary syphilitic affections, in order that we may afford greater space for this veteran's observations on the treatment, a subject now of such doubt and disputation.

"It appears to me, (says Mr. Foot) that very often the difference in opinion, about the administration of mercury, arises from that antipathy which theorists have conceived, by now and then seeing the baneful effects of mercury, in cases where it has been improperly gone on with."

He therefore justly observes, that although the power of mercury be always the same, yet, as every constitution possesses its own peculiar idiosyncrasy, and consequently the venereal symptoms are more or less rapid, malignant, and obstinate in one subject than another, so "it cannot, from these causes, be expected that an exact proportion of mercury will ever be ascertained, which, by curing one subject, will thereby be the standard in truth of that proportion which may be necessary for curing another."

"It is necessary for mercury to be pressed forward to a certain point; it is to be wished, that the point may be obtained without any bad constitutional effects. It is necessary that the venereal symptoms be extinguished, and for that end, that certain effects, arising out of the operation of mercury on the constitution, must come forward: it is to be wished, that as soon as these effects do come forward, the venereal symptoms may retire, and that the constitution be left with this specific difference, in consequence of the exchange of a mineral poison for a morbid one—that the mineral effect will cease, when the application of it be withdrawn from the constitution; but that the morbid effect will never cease, until the operation of mercury has gone on to a certain acmé, and until it has brought about this great and necessary revolution." 279.

Mr. Foot having very faithfully pourtrayed the symptoms attendant on mercury being carried to a great excess, which he does not advise; yet he observes,

"It is necessary that there should be a putrescent diathesis, fœtid breath, prostration of strength, affection of the gums, and increase of saliva; and that these appearances should be supported for a considerable time, even longer than the venereal symptoms remain, in order to eradicate the disease from the constitution. This may be almost said to be that desirable point of mercurial operation upon the constitution, which is calculated to effect every purpose of eradicating venereal symptoms. Without these mercurial appearances, without these positive inferences, that the whole of the fluids have undergone this necessary mercurial change, and have been continued for some time, by a regular succession of mercurial supply, I should not expect that a fixed, obstinate, and inveterate venereal infection could be eradicated." 290.

As the mercurial-symptoms are only kept up by a constant supply of mercury to the constitution, "so, whenever that supply is withdrawn, it is wonderful to see how the effects decline, especially if the patient change his situation to pure air, cleanse his body by a tepid bath, take that salutary restorative, nitric acid, and gentle exercise in the open air: in the course of three weeks there will not remain one solitary trace of mercurial symptoms."

Mr. Foot judiciously enjoins "confinement to the chamber, a regular diet, a *gradual* introduction of mercury into the constitution by friction, an exclusion of fresh air." "The cure, he observes, consists in persevering with a regular supply of mercury, until the venereal symptoms be extinguished"—"the mercury must not therefore be hurried into the constitution." Our author very justly condemns the alterative plan of curing syphilis by small doses of blue pill or calomel, conjoined with sarsaparilla, allowing the patient to go about in the open air. "I may venture (says he) to declare it as my opinion, that the mouth should be somewhat materially affected, and that affection continued for some time after a venereal chancre has healed." 300.

In these sentiments we entirely agree. Mr. Foot censures the daily use of the warm bath during the mercurial course. But if the ptyalism run too high, "the warm bath, nitric acid, and opening medicines, are essential means for reducing exacerbated symptoms from mercury." 301.

Mr Foot prefers, in general, inunction, as the best mode of administering mercury. "In scrofulous habits, it is more prudent to try a solution of sublimate in decoction of sarsaparilla."*

"Whether it be prudent to attempt a continuance of mercury, in any form, in a constitution thus conditioned, and when such is the result of its application, that must depend upon the necessity and pressure of the occasion. If the symptoms of venereal virus be ra-

* "Practitioners (says Mr. Foot) in England and Europe, are indebted to Dr. Scott for his Report of this valuable medicine, the result of his practice in the East Indies. It can be given when mercury cannot; and afterwards, mercury can be given with success." 301.

pid, either locally or constitutionally—if there be no time to be lost—if there be an immediate necessity to put a stop to them, an endeavour must certainly be made, by every possible means, to excite a complete mercurial stimulus, as the onfy means for effecting that end. But I believe, that in many instances, the effort will be vain, and the end cannot be obtained: and I know, and I feel, that such conditions are the most nice, dangerous, and intricate, of any which the disease and the remedy are exposed to. When mercury can be dispensed with for a time, it certainly ought to be; and pure air, milk diet, with light nutritious food and gentle exercise, must be the intervening resources. Nitric acid, bark, and steel, will be found to be the truest medicines for the purpose." P. 305.

We hope and trust, for the good of humanity, that the investigation now carrying forward by our brethren in the army, will develope sufficient evidence, that in cases of the above description, confinement, low diet, antimonials, &c. may arrest, or completely neutralize the syphilitic poison, so as to render it innocuous to the constitution, even without any mercury. We do not, however, anticipate, that the non-mercurial plan will ever apply generally in private practice. The investigation alluded to, however, will confer a lasting benefit on mankind, if it prove, that by any process, we can check syphilis in certain constitutions, without that mineral which has hitherto been considered the only specific for that purpose.

Our limits will not permit us to go into any farther details, nor is the work of that description which demands a regular analysis. But we cannot take leave of this veteran brother, without tendering him the homage of our respect and esteem. Throughout the whole of the work, which we have perused with attention, we see little to censure, and much to praise. We believe too, that were Mr. Foot's directions strictly followed, we should rarely meet with any of those victims to the poison of syphilis, or the poison, as it is called of mercury, which are held up, in terrorem, to paralyse our arm in the administration of the remedy, or distract our judgment as to the nature and treatment of the disease.

---

### § V. SKIN.

XIV. *Purpura Hæmorrhagica.** The two cases published by Dr. Parry of Bath, with successful issue, attracted the attention of the profession to a disease which, though not of frequent occurrence, was generally fatal under the old and favourite system of "tonics and nourishing diet."

"G. D. ætat. 18, of a pale, sallow complexion, and phlegmatic temperament, applied to me, September 7, 1819, with several petechiæ on the arms and breast. No pyrexia; pulse 100; bowels

* Case of Purpura Hæmorrhagica. By W. S. G. Davies, Esq. of Broad Street, Golden Square.

regular. Mittatur sang. ad ℥xx; this produced syncope. ℞ Nit. potass ʒß; jal. rad. pulv. ʒj; ℳ. et in chart vj divid. quarum sumat j. ter in die. This was on Tuesday morning, soon after its first appearance; and here it may not be amiss to mention what seemed to be the *predisposing and exciting causes.* He went the preceding evening to Bartholomew fair, where he walked about till he felt himself much fatigued; he went into the tap-room of a public house, without drinking, laid his head upon the table and slept; he woke about six o'clock, which was the hour he usually went to work; fearing from the long distance he had to go, he should not be in time, he ran from Smithfield to his master's house in Poland Street, without halting. On his arrival, after this exertion and violent exercise, complained of great thirst, and drank, notwithstanding the remonstrance of advisers, draughts of cold water. A few hours subsequently, the spots came out. 8th. The effusions under the skin increased, and extending over the body and inferior extremities; general uneasiness; no particular symptom, except quickness of pulse; had three or four stools from the aperient powders. He was desired to continue them, and to visit me next morning. From this time, till the following Sunday, the 12th, I saw no more of him; when I was called upon by his relation to go and see him. In the interim, the lad had been advised to apply at the Gerrard Street Dispensary, which he did; a letter of recommendation having been obtained for him. During this period he got worse, and was obliged now to keep his bed. The petechiæ were very thick, except upon the face; he had profuse hæmorrhage from the stomach, and oozing of blood from the ears, nose, gums, &c.; some pain of the head; slight coma; confined bowels; skin hot and dry; pulse 104; urine natural. V. S. rept.; could only get six ounces from the orifice. ℞ Hydr. submur. gr. viij; jal. rad. pulv. gr. xxiv; muc. acaciæ qs. ft. pil viij; st. ij. cum cochl. larg. iv. 4ta. q.h. mist. sequentis. ℞ Inf. sen. ℥vj; mag. sulph. ʒx; aq. menth. v. ℥ij ℳ. Diet, gruel and tea only, luke warm. 13th. No evacuation; complains of great giddiness; vomiting of blood, both dark and florid; the former for the most part. Heat of skin natural; pulse 108, hard, and firm; pain and swelling of the parotid and submaxillary glands, particularly of the left. Appl. hirud. et foment aq. calid. Enema domesticum; rept. mist. et pil 2ndo q.h. donec alv. copiose respond. 14th. Only two stools, dark and offensive; less giddiness; skin and tongue natural; no alteration of the petechiæ, nor of the hæmatemesis. The quantity of blood evacuated by the mouth since the 12th, about two quarts daily. Pulse 120, hard, and peculiarly irritable; this led me to suspect the chest, where the action of the heart was also strikingly obvious, beating against the cartilages of the ribs and sternum; V. S. rept. ad ℥xx. The blood, as in the former instance, had a firm appearance, with the addition in this of a spot of buffy coat, about the size of a sixpence. The most remarkable thing which struck my mind was the very small quantity of serum, not amounting to more than a tea spoonful in either of the large blood-lettings. ℞ Magnes. sulph. ʒiij; inf. rosæ ℥vj; tr. digit. ℳ xxiv. M. partem 3tiam. 8va. quaq. h.

15th. Pulse undiminished in frequency, hardness, or irritability; vomiting of blood stopt; tinitus aurium, but stupor and giddiness removed; more tumefaction of the lower jaw.—Rept. hirudines et foment. Rep. mist. ut heri.

16th. Ptyalism commencing; mouth very sore; three or four stools, dark and fœtid.—adde mist. tinct. digit. ♏ vj. Garg. pro ore.

17th. Petechiæ lighter; tumour of the left sub-maxillary gland increased. P. same.—Rept. hirudines et foment.; ten drops more added to the tincture.

18th. Three or four stools; face and neck much swelled; a moderate flow of saliva.—Twelve more leeches applied to the tumour. The mixture to contain 45 minims of the tinct. digit.

19th. Spots fading; pulse 128. Two or three stools of better appearance. Ulceration in the sublingual glands.—Repeat the mixture, with 15 ♏ plus tinct. digit.

20th. Pulse 120, with its peculiar vibration. Three or four motions. Sleeps well. Tendency to suppuration in the tumour.—Lintseed cataplasms to be applied often. The mixture to contain 65 ♏ of the digit.

21st. Pulse 128. Fluctuation of the tumour, opened and discharged about an oz. of thick pus. Spots fainter.—Rept. med.

22d. Pulse 136.—The mixture to contain 70 ♏ of the tincture.

23d. Pulse 132; bowels open; urine natural.—Rept. med.

24th. Despairing of having any control over the circulation, I diminished the fox-glove in order to abandon it, as the most prominent symptoms were the quickness of pulse with debility. Inf. cascarill. ℥ij ter in die.*

27th. Mouth better; pulse 126; tumour lessening; discharge moderate.

29th. Sat up; mouth better; eagerness for food. To be allowed light animal broths.

October 3d. Convalescent. Left off medicine.

10th. Went into the country.

Query 1st. Was not the accidental effect of the mercury on the constitution useful? and would it not, in similar cases, be a powerful auxiliary to the lancet?

Query 2d. How is the small proportion of serum in the blood to be accounted for?

Many other questions may arise from a perusal of the above statement."

---

* The tinct. digit. was obtained from Apothecaries' Hall.

XV. *Elephantiasis.** Mr. Robinson conceives that two distinct varieties, if not different diseases, are confounded under one name; "and what is worse, are treated alike, though they require very different remedies." As elephantiasis, the lepra arabum, is one of the most common, as well as "one of the most gigantic and incurable diseases" of Hindostan, we shall present a full analysis of Mr. Robinson's paper in this place, as it will thereby have a considerable circulation through our oriental and occidental dominions.

*Variety 1st.* Exhibits the following symptoms. One or two circumscribed patches appear upon the skin, (generally the feet or hands, but sometimes the trunk or face,) of a rather lighter colour than the neighbouring parts, neither raised nor depressed, shining and wrinkled, the furrows not coinciding with the lines of the contiguous sound cuticle. The skin of these patches is insensible, even to a hot iron. They spread slowly until the skin of the legs, arms, and whole body, is completely involved, and deprived of sensibility. It is in this state, chiefly, that the disease is remediable.

After a period, varying from two months to five or six years, symptoms indicative of internal disease, or functional derangement, are developed. The pulse becomes slow and heavy, the bowels torpid, the toes and fingers numbed, as with frost, appearing glazed, somewhat swelled, and nearly inflexible. The mind exhibits corresponding traits of torpor and inactivity; the soles of the feet and palms of the hands crack into fissures, dry and hard, as the parched soil of the country, the extremities of the toes and fingers, under the nails, being encrusted with a furfuraceous substance, and the nails themselves raised up until absorption and ulceration occur. Still there is no pain. The legs and arms now swell, the skin is every where cracked and rough, cotemporary with which symptoms, ulcers appear at the inside of the joints of the toes and fingers, directly under the last joint of the metatarsal or metacarpal bones; or they corrode the thick sole, under the joint of the os calcis or os cuboides, without any preceding tumour, suppuration, or pain, but apparently from simple sloughing off of the integuments, in layers of half an inch in diameter. A sanious discharge comes on; the muscles, in their turn, are destroyed; and the joint being penetrated as by an augre, "the extremity droops, and at length falls a victim to this cruel, tardy, but certain poison." The wound then heals, and other joints are attacked in succession, every revolving year bringing with it a trophy of this slow march of death! The patient, though a spectacle of horror to others, and a burthen to himself, still clings to life, and endeavours to cherish its remaining spark, by voraciously devouring all he can procure. "He will often crawl about with little but his trunk remaining, until old age comes on, and at last he is carried off by diarrhœa or dysentery,

* On the Elephantiasis, as it appears in Hindostan. By JAMES ROBINSON, Esq. Superintendant of the Insane Hospital at Calcutta. Medico-Chirurgical Transactions, Vol. x.

which the enfeebled constitution has no stamina to resist." Although the general health and the digestive functions do not suffer much throughout this long and tedious dismemberment, yet "a sleepy inertness overpowers every faculty, and seems to benumb, almost annihilate, every passion, as well of the soul as of the body, leaving only sufficient sense and activity to crawl through the routine of existence." This, our author considers as a distinct variety of elephantiasis, to which, on account of its most prominent trait, he would give the name of elephantiasis anaisthetos. He has never seen the larger joints attacked, (a strange assertion after telling us that the patient creeps about with "little but his trunk remaining") the nose destroyed, or any bones affected, save those of the hands and feet. The tuberculated species, hereafter to be described, sometimes supervenes, "but is by no means connected with, caused by, or necessarily subsequent to this disease."

*Treatment.* If we see the patient in the first stage, before described, the prognosis may be favourable. A combination of mercury and antimony, with topical stimulants, will generally succeed. A blister alone, kept open for a few days, will often restore the sensibility of the skin, and check the disease.

"Whenever the foot or hand alone is affected, I usually apply a strip of blistering plaster one inch and a half wide, all round the limb, just upon the line which marks the sound from the affected parts. Where this is inapplicable, from the extent of the disease, I apply a solution of muriate of mercury, made as follows:

℞. Hydr. muriat. gr. viij; acid. muriat. gt. xx. Tere in vit. mort. deinde adde spt. vini rectif. ℥ß aq. font. Oij. M. This must be rubbed well on the skin, wherever affected."

Mr. R. at the same time, gives to an adult, half a grain of calomel, three grains of antimonial powder, and from six to ten grains of rad. asclepiæ giganteæ every eight hours. This last medicine was discovered several years ago by Mr. Playfair, and our author thinks the professional world greatly indebted for the discovery of "the most valuable medicine hitherto derived from the vegetable kingdom." Mr. Playfair emphatically describes it as "a vegetable mercury, specific in the cure of lues venerea, leprosy, and cutaneous eruptions in general, the most powerful alterative hitherto known, and an excellent deobstruent. In all affections of the skin, says he, I have found it very effectual; and in the jugaru or leprosy of the joints, I have never failed to heal up all the ulcers, and often have produced a perfect cure."

In the complaint under consideration, Mr. Robinson agrees with Mr. Playfair, that the asclepias, called in Hindostan "*Mudar,*" is possessed of great virtues. He can also bear witness to its powerful effects as a deobstruent and sudorific in almost all cutaneous eruptions, arising from obstructed perspiration, and an apathy of the extreme vessels. It causes a sense of heat in the stomach, which rapidly pervades every part of the system, and produces a titillating feel upon the skin from the renewed circulation through the minute vessels. It is inadmissible where the affection is inflammatory, or the erruption pustular. Mr. R. tried it freely in lues venerea, but cannot venture to recommend it as a substitute for mercury. "It will enable you to heal a chancre, but does not eradicate the poison." In secondary symptoms, he considers it an admirable ally. Where mercury has been used, but cannot be safely pushed farther, the *Mudar* rapidly recruits the constitution, heals the ulcers, removes the blotches from the skin, and perfects the cure. The bark of the root is the only part of the plant that is useful in medicine, and should be gathered in March, April, or May. The bark, when well dried, is easily beaten into a fine powder, of which the dose is from three to ten grains. It grows in great plenty and wild throughout Hindostan.

*Variety 2d* Mr. Robinson would denominate elephantiasis tuberculata, which has been often described, and is now occasionally seen in this country. A very exquisite specimen was lately exhibited at Edinburgh, a plate and case of which is given in the Monthly Series of the Medico-Chirurgical Journal, by Dr. Lee. We shall not, therefore, copy Mr. Robinson's description of the disease, as he draws his delineation principally from the late Dr. Adams, and Dr. Bateman. In the tuberculated variety, the asclepias does harm; and is therefore inadmissible. Arsenic, in small doses, is the most useful medicine our author has found, but it is very far from being generally effectual.

Upon the whole, this is an interesting paper; and Mr. Robinson is intitled to the thanks of the profession for having made known to them a vegetable possessed of such valuable properties as he ascribes to the asclepias gigantea.

XVI. *Eczema Rubrum.** A female, aged 34, was admitted into the hospital, with a copious discharge of thin yellowish fœtid ichor, from almost the whole surface of the body, the cuticle scaling off, leaving the surface beneath of a red colour. Appetite good; pulse 110; tongue clean; bowels loose; catamenia irregular. She had been taking pills for a cough, about three months before, supposed to be mercurial, though they never affected her mouth, and during that period caught cold. The eruption then broke out. The ungt. cetacei applied to the parts—decoct. cinchonæ internally; the tepid bath. Subsequently, the cerat. plumb. super. was applied, and the sulphuric acid prescribed internally. Under this treatment, she recovered in about three or four months, but relapsed as bad as ever, all on a sudden. An ulcer on her leg had been dressed with ungt. nitrat. with some of which she had secretly anointed the axillæ, which were a little hard and rough. On closely observing the eruption this time, it had the appearance of innumerable minute vesicles, which, after two or three days, discharged a thin yellowish ichor, eroding the skin, and reducing the patient to the state before de-

* Case of Eczema Rubrum, with Remarks. By Dr. Crawford, Jun. M. D. of the Hampshire County Hospital. *Ed. Journal*, 62.

scribed. Linimentum aquæ calcis to the parts; citric acid internally; and when the irritation was abated, the tepid bath. In sixteen days she was well. Soon after this a dose of opium reproduced the disease. Dr. Moriarty mentions a case in which the eruption was also reproduced by opium three or four times.

Dr. Crawford justly doubts the effects of the *internal* medicines in this case. The last relapse was of shortest duration, although nothing but the tepid bath was employed. This eruption, especially in its primary attacks, appears most commonly to be owing to the action of mercury on a peculiar idiosyncrasy of constitution. Relapses often occur from other medicinal substances. "As this eruption puts on a *vesicular* form, the appellations of mercurial erythema or lepra, are inapplicable to it; and as mercury is not the only exciting cause, it is improper to denominate it hydrargyria or eczema mercuriale"*

---

### § VII. NERVOUS SYSTEM.

XVII. *Chorea.** Chorea appears to be a disease engrafted on the human constitution, like many other of the neuroses, by modern refinement and civilization. The *scelotyrbe* which Pliny [*lib. xxv. c. iii.*] describes as affecting the army of Germanicus on the Rhine, and which he attributes to unwholesome water used for drink, was supposed by Sauvages to be the disease in question, but the analogy of the two affections is too loose to warrant the conclusion drawn. It was not till the close of the sixteenth century, in fact, that chorea was correctly described, by Plater, Horstius, and Sennert. It is now well known, for it is far from being uncommon, though its pathology and treatment are by no means settled. We are of opinion that the neglect of viewing it under its idiopathic and symptomatic characters distinctively, has contributed to the diversity of sentiment respecting its nature and treatment. The idiopathic chorea is that form of the disease which is neither the symptom nor the effect of any other malady, and which generally appears about the epoch of puberty. Its predisposing causes are, the revolution in the system at the period alluded to, in subjects of feeble constitutions from infancy, and whose parents have been of nervous and irritable dispositions. Its exciting causes may very frequently be traced to mental agitations, as frights, violent fits of anger, the depressing passions, and certain affections of the genital system.

---

* We are happy to find that a Dispensary for Diseases of the Skin has been instituted in this metropolis, under most respectable auspices, and with the advantages of such able medical officers as Dr. Thomson, Dr. Emery, Mr. Wadd, Mr. Carpue, &c. Such an institution deserves the patronage of the profession, and the gratitude of the public.

* On the Use of Arsenic in the Cure of Chorea, By Mr. SALTER, Surgeon of Poole.—Medico-Chirurgical Transactions, vol. x.

The symptomatic chorea, offering nearly the same phenomena as the idiopathic, is dependent on a variety of local affections which should be carefully investigated; as too great fullness of the cerebral vessels, or the result of injuries of the head, irritation in some portion of the primæ viæ, or organs connected with digestion, suppressed eruptions, or general derangement of the nervous system from various mental or physical causes.

Any person who takes the trouble to reflect on these circumstances in the etiology of the complaint, will readily perceive that no one method of cure can be successful in all, or even in a majority of cases. Thus, when chorea appears as an idiopathic affection about the epoch of puberty in a plethoric girl, the practice of Sydenham, repeated bleeding and purging, will be the most efficacious treatment. If the patient be of a delicate, irritable, and what is termed a nervous disposition, then tonics, especially the oxydes of zinc and bismuth, or arsenic, with the fœtid gums and eccoprotics, will be the treatment indicated. If chorea depends on irritation or malsecretion in the line of the digestive organs, a continued train of purgatives, on the plan of Dr. Hamilton, will be useful. We may here state, however, what we believe a great number of practitioners could substantiate, that purgatives are not even *generally* successful in the complaint under consideration, when trusted to alone. We have been so often disappointed, in following up Dr. Hamilton's directions, that we have long since laid them aside, except as auxiliaries. From the oxyde of zinc, indeed, with alteratives and antispasmodics, we have derived, upon the whole, the most satisfactory results. But the etiology of the disease, as was before observed, should be particularly sought after, and we can confidently assure the practitioner, that the labour of his investigation will be amply repaid in the facility and rapidity of the cure. After this short, but we hope not irrelevant preliminary, we shall come at once to the subject of this article.

Soon after reading the account of a case of chorea cured by arsenic, in the 4th vol. of the Medico-Chirurgical Transactions, Mr. Salter had an opportunity of treating a case of the disease. He was unsuccessful with the purgative plan, and therefore "was induced to make trial of the liquor arsenicalis, and had soon the happiness to find his patient rapidly recover." In this case, however, he used the pil. gambog. compos. and therefore the advocates of purgation may attribute a great share of the success to the purgative medicine. But in the three cases detailed in the volume before us, "no other medicine was used than the liquor arsenicalis, nor was any change of regimen allowed; its efficacy, therefore, in them, must be considered as incontrovertibly established."

Case 1, Eliz. Hardman, ætat. 17, dark complexion, eyes, and hair, had chorea for three months. The members and trunk were affected; and indeed, the whole body was frequently thrown into the most grotesque attitudes. The bowels were constipated; the catamenia regular. After four days of purging, the complaint was rather increased than meliorated.

Four minims of the liq. arsenicalis were then ordered thrice a day. In two days the involuntary motions were somewhat less violent. The dose to be increased by one drop each time per diem. In ten days more, the patient was so much better that she appeared to be rapidly getting well. Fourteen drops of the liq. ars. were now taken thrice a day, the dose not to be increased. In a fortnight, this treatment removed the chorea, and greatly improved the general state of the patient's health. Griffith's mixture, with pil. aloes, cum myrrh, completed the cure.

Case 2. Miss P——, a delicate girl, of nine years, became affected with chorea, immediately after being frightened by one of her school-fellows. The free use of purgatives, with the decoct. cinchonæ, was tried for nearly three weeks, without the least success; on the contrary, the symptoms were daily augmenting in severity. The function of speech was nearly suspended. She now commenced with three drops of the liq. arsen. thrice a-day. In a month of this treatment, a striking impression had been made on the complaint, and in a few days more, the chorea disappeared entirely. A slight relapse was again cured by the arsenic.

Case 3. Mary Brown, ætat. 12, delicate constitution, had long been affected with chorea; but, during the last six weeks, it had been violent. She began with three drops thrice a day, increasing the dose one drop every day, until as much is taken as the stomach will bear. In three days she was better, and in less than a month she was cured.

These cases are interesting. We think that Mr. Salter was somewhat bold in the first case. Fourteen drops thrice a day, for more than a fortnight, is more than we would like to exhibit of the liq. arsenicalis, though few have a more favourable opinion of the medicine than we have. We consider it, in fact, the most powerful tonic in the Pharmacopœia, and we have given it in a very great variety of chronic and anomalous affections, with the most decided benefit. We have not seen any injurious effects result from its use, when not carried farther than eight or ten drops thrice a day, and never continued longer than a fortnight at one time. But we have seen a few, and heard of several cases, where a longer continuance of the medicine was injurious to the stomach.

---

### § VIII. GENERAL SYSTEM.

XVIII. *Tetanus*.* This most formidable disease has been too justly termed the opprobrium medicorum. It is, indeed, one of those which often reduce the practitioner to a painful dilemma; for he must

---

† A Case of Traumatic Tetanus, successfully treated; by Duncan Stewart, M. D. Surgeon in Chief to the King of Hayti; with Remarks by J. Allan, Esq. Surgeon, London.

either incur the risk of killing his patient, or see him carried off amidst the most unrelenting and frightful tortures.

The following case, in which it was successfully treated in its most dangerous form, affords decided evidence of Dr. Stewart's practical skill, and leads us to expect results equally beneficial to his patients and creditable to himself, from the cultivation of other departments of that extended field in which he is now exerting his ingenuity and his talents. This account, which is merely extracted from a private letter, contains only the leading features of the case; but these, as well as the judicious means by which the cure was accomplished, are so clearly, though briefly described, as to merit the attention of the medical public.

*Case.* The patient was brought to the public hospital at Cape Henry, with a large splinter of wood in the sole of his foot; his jaw considerably locked; the muscles on the back of his neck and trunk so strongly contracted as to form the spine into a complete arch, and his extremities perfectly rigid. After removing the splinter, Dr. Stewart began the treatment by giving two ounces of the oleum terebinthinæ, mixed with one ounce of castor oil, twice a day. This plan was continued for five days; during which, glysters, containing each an ounce of the oleum terebinthinæ, were exhibited three times a day. During the first day, two grains of opium were given every three hours; during the second day, two grains every two hours; during the third day, two grains every hour; and during the fourth, fifth, sixth, and seventh days, the same dose was repeated every half hour. The patient took two bottles of Claret daily. A large blister was applied to the pit of the stomach on account of local pain. By the oleum terebinthinæ and castor oil, he was freely purged every day; an operation which, as Dr. Stewart remarked, was promoted by the opium. After the fifth day, the fits of spasm became less frequent; and, for the first time after his admittance, the patient enjoyed some sleep. After that period too, the opium was the only medicine given, and yet the purging continued regularly, and was, by Dr. Stewart, ascribed unequivocally to that medicine. When a decided amendment of the symptoms had become apparent, the doses of it were gradually decreased. On the tenth day, the patient was quite free from the disease; and what is very remarkable, one grain of opium twice a day *then* occasioned head-ach, thirst, and giddiness. During the process of cure, the wound was dressed with simple poultices.

Dr. Stewart adds, that several less severe cases had yielded more readily to the same plan of treatment; and it is by no means intended here to anticipate any observations on this disease which he may probably hereafter communicate to the public; but it may be useful at present to compare with the plan adopted by Dr. Stewart the observations contained in the following extract from the Treatise of Dr. Morrison on this subject, as it appears at page 375, in Dr. Johnson's work on Tropical Climates. On this comparison, it will be found, that in resorting to the oleum terebinthinæ, and freely purging the bowels, Dr. Stewart effectually accomplished what Dr. Morrison considers a great desideratum, but exceedingly difficult to be effected. The remark made by Dr. Stewart relative to the operation of opium, in promoting catharsis, is also conformable with Dr. Morrison's experience.

"The bowels (says this gentleman) should be kept as free as possible. We must endeavour to bring about an operation every twelve hours. This, even by the aid of strong cathartics, or purgative injections, will be found very difficult to be obtained, the sphincter ani sometimes scarcely admitting the introduction of a glyster-pipe, and the exhibition of the strongest purgatives may often be attended with little or no effect. Sulphate of soda, jalap and calomel, scammony, pil. aloës, cum colocynthide, &c. are as proper for this purpose as any other, aided by stimulating glysters, such as solutions of muriate or sulphate of soda, with olive oil; the resin of turpentine suspended by the yolk of an egg, solutions of soap, &c. &c. I have found it, on two or three occasions, impossible to open the bowels freely, till after large quantities of opium had been taken, which seemed to bring about a general relaxation." Dr. Morrison once gave a patient, who ultimately recovered, ten grains of opium and twenty of calomel in pills, and five ounces of tincture of opium in wine, within the space of twelve hours.

"As next in importance to opium, Dr. Morrison recommends the preparations of mercury in large doses internally, conjoined with a free use of frictions with the ointment externally, and with these he recommends that liberal, and, indeed, great quantities of wine and ardent spirits should be given."

Dr. Johnson states his opinion, that "tetanus is radically an inflammatory disease," and the writer of this article could confirm the accuracy of this opinion, by the detail of a case in which the disease was cured by bleedings, repeated to the extent of above one hundred and thirty ounces, active purging with senna and salts, and the application of blisters. Nevertheless, Dr. Johnson justly remarks,* "that the local abstractions of blood by leeches, and cupping from the neighbourhood of the spine, with subsequent blisters there, are not inconsistent with the plan of treatment recommended by Dr. Morrison. For it must be remembered, that such is the unequal distribution both of the blood and excitability in the system, under this disease, that one part is completely torpid, while another is on the point of extravasation from turgescence or inflammation. It is evident, from this view of the affair, that we must stimulate the torpid organs at the very moment we are employing sedatives and counter-irritants, or abstracting blood from the congested parts. Hence, too, the great value of purgatives and mercury. The former bring back the excitement to the abdominal viscera, and powerfully determine from the spine: the latter sets all the secretory and

---

* Tropical Climates, p. 376.

excretory apparatus to work, while it equalizes the circulation in every part of the system."

The still more simple plan of treatment acted upon by Dr. Stewart, as far as it has been tried, seems, without the aid of mercury, to promise the accomplishment of all the intentions proposed by Drs. Morrison and Johnson, merely by the well directed and vigorous application of three well known medicines, oleum terebinthinæ, opium, and castor oil.

---

### § IX. MEDICINAL AGENTS.*

XIX. *Portable Sudatorium.* † The modest little Pamphlet whose Title is subjoined, deserves the patronage of the Profession, as containing several ingenious and practically useful contrivances auxiliary to the healing Art. We have selected the *sudatorium*, as a specimen; recommending our brethren to purchase the pamphlet itself, in order to avail themselves of the plates, in the construction of the auxiliaries in question.

The sudatory cradle is made of longitudinal bars of osier, placed at the distance of an inch or more asunder, and preserved in their situations by occasional cross-bands of basket-work. Its length is about four feet; and main width, at the base, which covers the patient's shoulders, two feet—the smaller end about a foot and a half. It may thus be compared to the half of a truncated cone, divided in a direction from the apex to the base. Or it may be compared to the cradle which is placed over a fractured limb, to keep off the weight of the bed-clothes. Within the edges of the narrow end is laced a thin piece of board, with a circular orifice in its centre, for the admission of the point of a curved tube, through which the warm vapour is to be conveyed. This curved tube is composed of sheets of tin-plate, grooved, but not soldered, to prevent the joints from being separated by the heat of the lamp placed under its wider extremity. The tube is about thirteen inches long; the larger opening or end is three inches in width; the smaller, one inch and a half. It is in shape resembling a powder horn. It has a few holes in its sides, near the wide extremity, to admit the cool air from without, as the lamp rarefies the air within. The lamp itself ought to be grooved also, and not soldered, as it has a considerable degree of heat to bear. It is merely a spirit of wine lamp, with three burners.

---

* We have been obliged to throw out several Sections for want of room, till next Number.

† Auxiliaries to Medicine, in four Tracts; illustrated by Engravings on Wood and Copper. By Charles Gower, M. D. Fellow of the Royal College of Physicians, and Physican to the Middlesex Hospital, &c. Octavo, stitched, pp. 44, and five Plates. London, 1819.

The patient being placed in the easiest posture his feelings indicate, on his bed, and wrapt in a small blanket, the cradle is to be laid over him, and over the cradle the upper bed-clothes, carefully tucked in under the edges of the said cradle, leaving, of course, the head of the patient at liberty to be withdrawn from the cavity, and allowing the bed-clothes to be folded under the chin. "In this state of things, the attendant has nothing more to do than to pour two ounces, or less, of pure spirit of wine within the lamp, and to place it, lighted, under the curved tube at the foot of the cradle," having previously fixed the small end into the board at the foot of the cradle.

"In the space of a few minutes, (says Dr. Gower) the fresh air, which rushes in at certain apertures in the tube, is so heated and rarefied as to pass on rapidly into the cavity of the cradle, where the patient soon feels the warmth, and is shortly afterwards made to exude plentifully from every pore." 7.

We forgot to mention that the lamp must be made to fill up exactly the aperture of the tube, otherwise the heat will escape uselessly into the room.*

We need not take up any space with remarks on the great advantages resulting from a sudatory process in various diseases; but we conceive, that the profession at large is indebted to Dr. Gower for the promulgation of this very cheap, convenient, and useful *sudatorium.*

Our limits will not permit us to notice the other ingenious auxiliaries to medicine, delineated in this little pamphlet, but we trust that enough has been said to induce our Readers to consult the original.

XX. *Medicinal Springs.*† The waters of Harrogate owe not their celebrity to any fashionable, and, of course, capricious circumstance. They have recommended themselves by their own intrinsic and valuable properties; and, consequently, are every year, obtaining an increase of reputation. Dr. Hunter does not touch on the chemical or medicinal qualities of the original Harrogate Springs; but only of the two new ones. Those, therefore, who wish for a general account of these celebrated waters, must consult the 4th edition of Dr. Thomas Garnett's work, published in 1804.

The two springs in question were discovered about twelve months since, and are situated two hundred yards from the promenade room, with a north-eastern aspect, commanding a pleasant view of the neighbouring valley.

---

* A complete Sudatorium cradle, curved tube, lamp, and extinguisher may be had of Mr. Smith, No. 14, Gerard Street, Soho, for *Thirty Shillings.*

† An Essay on two Mineral Springs recently discovered at Harrogate; and on the Springs of Thorp-arch and Ilkley, &c. &c. By Adam Hunter, M.D. F.R. M.S. E. Physician to the House of Recovery at Leeds. Octavo, sewed, 134 pages. 1819.

1. *New Saline Chalybeate Spring.* When the water is first taken up, it is transparent, with rather a sparkling appearance, the taste saline, and not so unpleasant as the sulphur water—temperature 47. Specific gravity, 1,0075. Gaseous contents as follows: A wine gallon contains, carbonic acid gas, 6.320; azotic gas, 3.970; oxygen gas, 0,870, cubic inches. Saline ingredients in the same quantity as underneath.

| | Grains. |
|---|---|
| Muriate of soda - - - - - | 434.00 |
| Muriate of lime - - - - - | 30.00 |
| Muriate of magnesia - - - | 13.00 |
| Sulphate of lime - - - - | 9.00 |
| Carbonate of iron - - - - | 5.00 |
| Carbonate of lime - - - - | 3.00 |
| Loss - - - - - - - - - - - - | 2.50 |
| Total - - | 496.50 in a gallon. |

2. *New Chalybeate Spring.* The taste of this spring is distinctly and strongly chalybeate. The temperature was 48, when that of the surrounding atmosphere varied from 58, to 64. Specific gravity 1.0012.—A wine gallon contains

| | Grains. |
|---|---|
| Carbonate of iron - - - | 10.50 |
| Muriate of soda - - - - | 2.50 |
| Magnesia a very small quantity. | |

| | Cubic Inches. |
|---|---|
| Carbonic acid gas - - | 16.500 |
| Azotic gas - - - - - - | 4.200 |

Dr. Hunter conceives, that the saline chalybeate well, [1] holds a middle rank between the waters of Leamington Priors, and the saline chalybeate wells at Cheltenham; differing from the latter, in containing a larger proportion of the muriates, and less of the sulphates.

"From these considerations, as well as the acknowledged utility of the contents, we may anticipate that this spring will be a valuable addition to the Harrogate waters. And as many people, after having drunk the sulphur water, at Harrogate, for a few weeks, are recommended to proceed to Cheltenham, it might be desirable before they undertake this journey, to many so unpleasant and inconvenient, as well as expensive, that they should give this water a fair trial."

The water is gently aperient, and must be taken in considerable quantities for that purpose. The chalybeate spring, from the analysis, appears to contain a larger proportion of the carbonate of iron, than almost any water of the same class hitherto discovered. At the date of the work under Review, (July 25th, 1819) upwards of seventy invalids were taking the *saline chalybeate water*, "and with the most beneficial results." It may be taken with two different intentions, namely, as an evacuant, and as a gentle alterative, the quantity being regulated for that purpose. To produce a cathartic effect, half a pint should be taken, and repeated once or twice, at the interval of ten or fifteen minutes. It may, however, be taken to the amount of two or three pints, not only with safety but advantage.

The waters of Thorp-arch (beautifully situated upon the banks of the River Wharfe, twelve miles from Leeds, Harrogate, and York) do not differ materially from those of Harrogate, either in chemical or medicinal properties, and, therefore, are passed over.

The topography of Ilkley, a village situated on the south side of the Wharfe, about sixteen miles from Leeds, is pourtrayed by Dr. Hunter with great animation, and descriptive eloquence. The only accounts of its spring, that have ever been given to the public, are to be found in Dr. Mossman's Essay on Scrofula, and in Dr. Short's work; but there the chemical analysis is imperfect. Through the assistance of a very intelligent friend of the author's, Mr. Spence, surgeon, at Otley, who has had ample opportunities of observing the action of the water, Dr. H. has laid before the public, as the joint labours of himself and friend, an account, chemical and medicinal, of the Ilkley fountain, of which we shall state some of the particulars in this place.

In respect to its sensible properties, Dr. H. and Mr. S. inform us, that it possesses a crystal clearness, is devoid of taste or smell, and feels peculiarly grateful to the stomach. A wine gallon of the water contains, 6.50 grains muriate of lime; 3.00 muriate of magnesia; 12 cubic inches of carbonic acid gas, and 5 of atmospheric air. From this it appears, that the Ilkley spring is a very pure soft water. From the great depth of its course in the earth, its temperature is found to be 48 deg. This spring is principally resorted to for the purpose of bathing; though its internal use should not be lost sight of, since, when taken in considerable quantities, it produces an immediate determination to the kidnies, and its diuretic properties generally continue during the course.

Throughout this little work are scattered a great number of acute remarks and judicious observations, relative to the management of what are termed the non-naturals. But as they are directed more to the invalid than to the profession, they require no comment here. Dr. Hunter appears to us to be a man of warm feelings, generous sentiments, and vigorous mind. With such qualities and qualifications we augur well of his future professional career, and predict that other, and still better productions will yet issue from his pen.

